# Mendelian randomization and colocalization reveal potential causal effects of average daily gain on carcass composition and reproductive traits in pigs

**DOI:** 10.1186/s40104-026-01363-5

**Published:** 2026-04-02

**Authors:** Wondossen Ayalew, Guangzhen Li, Yuqiang Liu, Jinyan Teng, Xiaodian Cai, Qing Lin, Yahui Gao, Jiaqi Li, Zhe Zhang

**Affiliations:** State Key Laboratory of Swine and Poultry Breeding Industry, Guangdong Laboratory for Lingnan Modern Agriculture, Guangdong Provincial Key Lab of Agro-Animal Genomics and Molecular Breeding, College of Animal Science, South China Agricultural University, Guangzhou, 510642 China

**Keywords:** Causal inference, Functional genomics, Meta-GWAS, Pleiotropy, Swine

## Abstract

**Background:**

Selective breeding has substantially improved productive and reproductive traits in pigs. Yet, these traits are biologically interconnected, and selection for one often affects others in unintended ways. While genome-wide association studies (GWAS) have uncovered many loci linked to these traits, they provide limited insight into causal mechanisms. Mendelian randomization (MR) provides a robust framework for inferring causality and identifying shared genetic determinants. Here, we integrated MR, colocalization, and functional genomics to investigate the biological links between growth, carcass composition, and reproduction in pigs.

**Results:**

Using average daily gain (ADG) as the exposure, MR revealed potentially significant causal effects (*P* < 0.05) of ADG on carcass composition traits, including backfat thickness (BFT: $\widehat{b}{ }_{xy}$ = 0.13) and loin muscle depth (LMDEP: $\widehat{b}{ }_{xy}$= 0.35), suggesting a potential causal contribution of increased ADG to both fat deposition and muscle development. Additionally, ADG showed a negative causal association with age at first farrowing (AFF: $\widehat{b}{ }_{xy}$= −3.24), indicating that faster growth promotes earlier sexual maturity and reproductive onset. In addition to the statistical evidence, gene annotation of instrumental variants (IVs) identified overlapping candidate genes, which may help explain the causal associations observed across the four exposure–outcome analyses. Among these, *MC4R* and *CDH20* were supported by colocalization analysis, indicating shared genetic signals potentially linking growth, carcass composition, and reproductive traits. Integrative analysis supported the tissue-specific roles of these genes in regulating growth, carcass composition, and reproduction.

**Conclusions:**

Our findings suggest a shared genetic architecture and provide potential evidence of a causal influence of ADG on carcass composition and reproductive traits in pigs. This integrative framework supports the development of multi-trait breeding strategies that enhance productivity while managing inherent trade-offs in regulating complex traits.

**Supplementary Information:**

The online version contains supplementary material available at 10.1186/s40104-026-01363-5.

## Background

Genetic selection has been a cornerstone of livestock breeding, driving substantial improvements in economically important traits [1, 2]. Production and reproductive traits are fundamental determinants of overall efficiency and profitability in pig farming. These traits are genetically correlated, exhibiting both favorable and antagonistic relationships; for example, selection for faster growth can negatively affect reproductive performance [3, 4]. The genetic architecture of these traits is further shaped by complex interactions between genetic and environmental factors [5, 6]. While estimates of genetic correlations provide insight into the strength and direction of these relationships, they do not establish causality. Evidence from cattle [7] highlights that accounting for causal relationships between production and reproduction is crucial for optimizing productivity across livestock species.

Recent advances in GWAS have significantly improved our understanding of the genetic basis of complex traits in pigs, enabling the identification of loci associated with specific phenotypes [8, 9]. However, trait correlations may arise from direct causal effects, shared genetic architecture, or multiple forms of pleiotropy, complicating causal interpretation. Addressing these complexities requires causal inference approaches that move beyond standard association analyses. Randomized controlled trials (RCTs), in which individuals are randomly assigned to groups based on exposure status (e.g., treatment), remain the gold standard for establishing causality. RCTs minimize both known and unknown confounding factors, allowing observed differences in outcomes to be attributed to the exposure under investigation [10, 11]. However, in livestock breeding, especially for complex traits, RCTs are often impractical due to logistical and financial constraints [12].

Additionally, as noted by Schwarz et al. [7], controlled experiments are typically conducted under standardized conditions with limited sample sizes and genetic diversity, which may not adequately capture the variability present in commercial livestock systems. This limitation restricts the applicability of trial findings to real-world breeding programs. Thus, large-scale observational data reflecting the full complexity and diversity of commercial populations serve as the primary resource for studying genetic architectures and trait relationships in livestock.

Given these challenges, alternative approaches such as MR, which leverage the random allocation of alleles at conception as a natural experiment, have emerged as powerful tools for causal inference in livestock. MR uses genetic variants as instrumental variables (IVs) to estimate the causal effect of one trait on another, thereby reducing bias from confounding factors—including environmental, management, and biological influences, as well as reverse causation—provided its core assumptions are met [13, 14]. Unlike human studies, where large exposure cohorts often reflect self-determined behaviors such as alcohol consumption and smoking [15], livestock populations lack naturally varying exposures, which limits the identification of strong IVs and makes MR applications more challenging. Nevertheless, recent studies in dairy cattle have demonstrated their potential to uncover causal links between production traits and health or reproductive outcomes [7, 12]. To date, however, no comprehensive MR study has been conducted in pigs to elucidate causal relationships among growth, reproduction, and carcass composition traits, despite their central importance to breeding goals. Addressing this gap is crucial for enhancing genetic selection strategies by gaining a deeper understanding of the causal architecture underlying economically important traits.

Colocalization analysis provides a complementary approach to determine whether two traits share the same causal variant or genetic signal across loci [16, 17]. Integrating MR with colocalization enhances causal inference by ensuring that genetic signals influencing multiple traits arise from the same variant rather than from nearby linked variants [18]. Furthermore, given that a substantial proportion of GWAS-identified variants reside in regulatory regions [19, 20], incorporating functional genomic resources (e.g., FarmGTEx) is vital for interpreting their biological relevance [21]. The Pig Genotype-Tissue Expression (PigGTEx) project provides a comprehensive resource for characterizing the effects of genetic variants on gene expression across multiple tissues, facilitating the identification of regulatory mechanisms underlying economically important traits in pigs [22]. In this study, we integrate MR, colocalization, and functional genomics resources to investigate the causal genetic architecture of growth, carcass composition, and reproductive traits in pigs. This comprehensive analytical framework facilitates the identification of candidate causal genes and regulatory mechanisms, providing valuable targets for precision breeding and functional validation.

## Materials and methods

### GWAS summary statistics and study populations

We utilized meta-GWAS summary statistics generated from previous large-scale association studies conducted within our research group, which were deposited at the PigBiobank (https://pigbiobank.farmgtex.org; [23]). This study focused on five economically important traits in pigs: ADG, BFT, LMDEP, AFF, and the number of stillborn (NSB) piglets. Phenotypic records per trait ranged from 5,707 (AFF) to 39,323 (BFT) pigs, collected from Duroc, Landrace, and Yorkshire breeds. Genotyping was performed using medium-density porcine SNP genotyping arrays (Additional file 1: Table S1), followed by imputation to whole-genome sequence level using Beagle (v5.1) [24] and the multi-breed Pig Genomics Reference Panel (PGRP v1) developed within the PigGTEx project [22]. Only SNPs with minor allele frequency *P* > 0.01, call rate > 95%, and Hardy–Weinberg equilibrium $P>1\times 1{0}^{-6}$ were retained [9, 23]. After stringent quality control, the number of tested autosomal, biallelic SNPs per trait ranged from approximately 20 to 27 million (Table 1).

**Table 1:** Summary of GWAS datasets used for MR and colocalization analyses

Trait	Phenotypic records	Tested SNPs	Significant SNPs (${\boldsymbol{P}}<5\times 1{0}^{-8}$)	Individual GWAS studies
ADG	20,555	23,787,839	5,439	22
BFT	39,323	26,921,471	20,809	43
LMDEP	24,215	21,997,829	1,889	20
AFF	5,707	26,921,471	2	12
NSB	17,077	20,009,048	81	21

*ADG* Average daily gain, *BFT* Backfat thickness, *LMDEP* Loin muscle depth, *AFF* Age at first farrowing, *NBS* Number of stillborn

Genome-wide association analyses were performed separately for each trait and population using a mixed linear model implemented in GCTA-fastGWA (v1.94) [25], adjusting for sex, farm, year, and season as fixed effects. The first five genotype principal components were included to control for population structure [9, 23]. A total of 118 individual GWAS were performed (Table 1). Summary statistics from each GWAS were quality-controlled using EasyQC [26] and subsequently combined within each trait using inverse-variance weighted meta-analysis implemented in METAL (v2011-03-25) [27]. The meta-GWAS identified shared loci across populations, accounting for sample size and effect heterogeneity while controlling for genomic inflation [9, 23].

### Exposure and outcome definition

To better understand the interconnections between growth, carcass composition, and reproductive traits, we defined putative exposure and outcome traits. ADG was selected as the exposure trait, while BFT, LMDEP, AFF, and NSB were designated as outcomes. The selection of ADG reflects its documented genetic associations with carcass composition and reproductive traits, as well as its long-standing role as a target of selection in pig production for over a century. Strong positive genetic correlations between ADG and carcass composition traits (BFT and LMDEP) have been consistently reported [28, 29]. Additionally, ADG exhibits a favorable negative genetic correlation with AFF (−0.45) [28] and NSB (−0.38) [29], which are considered important in pig breeding. These established genetic connections provided the rationale for investigating ADG as an exposure, and carcass composition and reproductive traits as outcomes, within the MR framework. MR leverages genetic variants as IVs to infer the causal effect of an exposure on an outcome [30]. The fundamental principle is that, if the exposure (*x*) causally influences an outcome (*y*), then genetic variants (IVs, SNPs, and *z*) that are robustly associated with *x* should also show proportional effects on *y*, provided there is no horizontal pleiotropy. Under these assumptions, the estimated effect of *x* on *y* ($\widehat{b}{ }_{xy}$) should be consistent across independent IVs [30]. To implement this, IVs were selected from GWAS summary statistics based on a stringent genome-wide significance threshold $({P}_{GSMR}<0.05\times 1{0}^{-8})$, followed by LD clumping using the PGRP v1 reference panel [22] to ensure quasi-independence among SNPs. Clumping was performed with the default parameters described in Zhu et al. [30]. For each selected SNP, we calculated the proportion of variance explained (*R*^2^) and the F-statistic to assess the instrument's strength. SNPs with *F* < 10 were excluded to minimize weak instrument bias and ensure reliable causal estimates [13].

### MR analysis

Generalised Summary-data-based Mendelian Randomisation (GSMR) analysis was conducted using the GSMR2 package (v1.1.1) [31] implemented in GCTA (v1.94.0) [32]. This approach accounts for potential confounding from LD structure, sample overlap, and horizontal pleiotropy [30, 33]. For each instrumental SNP *i*, the causal effect of the exposure trait (ADG) on the outcome trait was estimated as the ratio of SNP effects:

$$\widehat{b}{ }_{xy\left(i\right)}=\frac{\widehat{b}{ }_{zy\left(i\right)}}{\widehat{b}{ }_{zx\left(i\right)}}$$

where $\widehat{b}{ }_{xy}$ and $\widehat{b}{ }_{zy}$ denotes the effect of the SNP on the exposure and outcome, respectively. When multiple independent or near-independent SNPs (*m*) were available, the overall causal estimate was obtained using an inverse-variance weighted (IVW) estimator:

$$\widehat b{}_{xy}=(1'V^{-1}1)^{-1}1'V^{-1}\widehat b{}_{xy},$$

where $\widehat{b}{ }_{xy}$ = {$\widehat{b}{ }_{xy(1)}$, $\widehat{b}{ }_{xy(2)}$,…$\widehat{b}{ }_{xy(m)}$}, $1$ is a *m* × 1 vector of ones, and $V$ is the variance–covariance matrix that accounts for LD between SNPs. Under standard assumptions $\widehat{b}{ }_{xy}$ follows a multivariate normal distribution with the variance $({1}{^\prime}{V}^{-1}1{)}^{-1}$.

To minimize bias from correlated SNPs and horizontal pleiotropy, instruments were subjected to LD clumping as described by Zhu et al. [30], ensuring a minimum of ten variants per trait. Horizontal pleiotropic variants were identified and excluded using the HEIDI-outlier test (${P}_{HEIDI}$ < 0.01) implemented in GSMR [30]. To account for multiple testing across the four outcome traits, we applied a Bonferroni correction using the number of tested traits (*n* = 4). Thus, the significance threshold was set at ${P}_{GSMR}<0.05/4$ = 0.0125.

### Annotation and candidate gene identification

SNPs used as IVs to infer causality were directly annotated to genes using the Ensembl Variant Effect Predictor (VEP, release 114) [34]. We did not apply flanking windows during annotation, thereby minimizing the inclusion of presumed linked genes and ensuring a more direct correspondence between IVs and annotated genes in the MR results. Gene coordinates were based on the *Sus scrofa* 11.1 genome assembly [35]. Only genes with validated and standardized gene symbols approved by the official nomenclature were retained [36]. Shared candidate genes associated with the four selected traits were subsequently visualized using the VennDiagram package (v1.7.3) in R v4.3.1 [37, 38].

### Colocalization analysis

To investigate whether ADG, carcass composition, and reproductive traits share common genetic association signals, we performed colocalization analysis using the coloc R package (v5.1.0) [16]. Genomic regions were defined as ± 500 kb windows around IVs of each exposure–outcome pair. Within these regions, we extracted GWAS summary statistics for ADG and the respective carcass composition and reproductive traits to generate region-specific datasets for colocalization analysis. This approach estimates the posterior probability that the same causal variant underlies associations for both traits within a region, providing statistical evidence to distinguish true shared genetic signals from coincidental associations driven by LD. Colocalization applied a Bayesian framework to evaluate five mutually exclusive hypotheses: no association with either trait (H0); association with only trait 1 (H1); association with only trait 2 (H2); association with both traits but driven by distinct causal variants (H3); and association with both traits sharing a common causal variant (H4). Default prior probabilities were used to represent the likelihood of each scenario before analyzing the data: ${P}_{1}=1\times 1{0}^{-4}$ (variant associated with trait 1), ${P}_{2}=1\times 1{0}^{-4}$ (variant associated with trait 2), and ${P}_{12}=1\times 1{0}^{-5}$ (variant associated with both traits). Posterior probabilities were then estimated, quantifying the probability of each hypothesis given the observed data. Strong evidence for colocalization was defined as a posterior probability for H4 (PP.H4) exceeding 0.9 [16]. This colocalization analysis served as a complementary approach to MR, aiming to identify loci potentially affecting multiple phenotypic traits through shared genetic signals.

To explore the functional relevance of candidate loci, genes located within ± 500 kb of the significant variants were annotated. The biological significance of these genes was evaluated using publicly available resources, including gene expression data from PigGTEx (https://piggtex.farmgtex.org/) and PubMed literature searches (https://pubmed.ncbi.nlm.nih.gov/). This proximity-based gene search identified loci where nearby genes may influence ADG, carcass composition, and reproductive traits, suggesting candidates for shared genetic architecture underlying these traits. We further assessed the potential functional impact of candidate variants using Ensembl Variant Effect Predictor (VEP release 114) [34]. We integrated tissue-specific chromatin state data from 14 pig tissues [39] to characterize the regulatory potential of colocalized regions. Visualization was performed using ggplot2 in R v4.3.1 [37].

### Integrative analysis with PigGTEx

Candidate genes associated with the traits of interest that overlapped between the annotated variants identified in the MR and colocalization analyses were further investigated for potential functional relevance using an integrative approach that combined expression quantitative trait loci (eQTL) mapping, transcriptome-wide association studies (TWAS), and phenome-wide association studies (PheWAS). Tissue-specific gene expression and association data were obtained from the PigGTEx [22] and PigBiobank [23] databases, which provide precomputed eQTL, TWAS, and PheWAS results across multiple pig tissues. Gene expression profiles across 34 pig tissues were examined using transcripts per million (TPM)–normalized data from the PigGTEx database. To further assess gene–trait relationships, each gene was individually queried in the PigBiobank database to retrieve precomputed TWAS results, enabling the identification of associations between predicted gene expression and complex traits. In parallel, precomputed PheWAS results from PigBiobank were used to explore broader phenotypic associations across biological systems. All results were visualized in R (v4.3.1) using the ggplot2 package [37] to support the interpretation of candidate genes implicated in growth, carcass composition, and reproductive traits.

## Results

### GSMR reveals trait-specific horizontal pleiotropy

The HEIDI outlier filtering excluded four variants with evidence of horizontal pleiotropy from the ADG and BFT analysis, and two from the ADG and LMDEP analysis. No horizontal pleiotropic variants were detected for either AFF or NSB. The filtered variants were distributed across different *Sus scrofa* chromosomes (SSC), with three variants on SSC1 and three on SSC7 (Table 2). Among the horizontal pleiotropic variants, those shared between ADG and BFT exhibited concordant effects, while two variants exhibited antagonistic effects between ADG and LMDEP on SSC7.

**Table 2:** Horizontal pleiotropic variants that were identified by the HEIDI method

CHR	Position, bp	ADG		Carcass composition traits		
		$\widehat{b}{ }_{xy} (SE)$	${P}_{GWAS}$	trait	$\widehat{b}{ }_{xy} (SE)$	${P}_{GWAS}$
1	158,058,352	−3.28 (0.58)	5.80 × 10⁻^1^	BFT	−0.14 (0.03)	6.41 × 10^−^^8^
1	162,719,373	−5.47 (0.81)	8.10 × 10⁻^1^	BFT	−0.21 (0.04)	7.35 × 10^−^^9^
1	162,899,224	−3.26 (0.59)	5.90 × 10⁻^1^	BFT	−0.17 (0.03)	2.15 × 10^−^^8^
7	29,132,104	2.33 (0.57)	5.70 × 10⁻^1^	BFT	0.14 (0.03)	6.66 × 10^−^^7^
7	21,813,401	6.63 (1.52)	1.37 × 10⁻^5^	LMDEP	−0.23 (0.07)	2.00 × 10^−^^3^
7	33,305,936	−3.20 (0.77)	3.27 × 10⁻^5^	LMDEP	0.45 (0.08)	8.24 × 10^−^^8^

Chromosomal location (CHR) and genomic position of the variants, the effect size ($\widehat{b}{ }_{xy})$ standard error (*SE*), and *P*-value (${P}_{GWAS}$) derived from GWAS summary statistics for exposure (ADG) and the outcome traits. *ADG* Average daily gain, *BFT *Backfat thickness, *LMDEP* Loin muscle depth

### Potential causal effects of ADG on carcass composition and reproductive traits

**Fig. 1:**
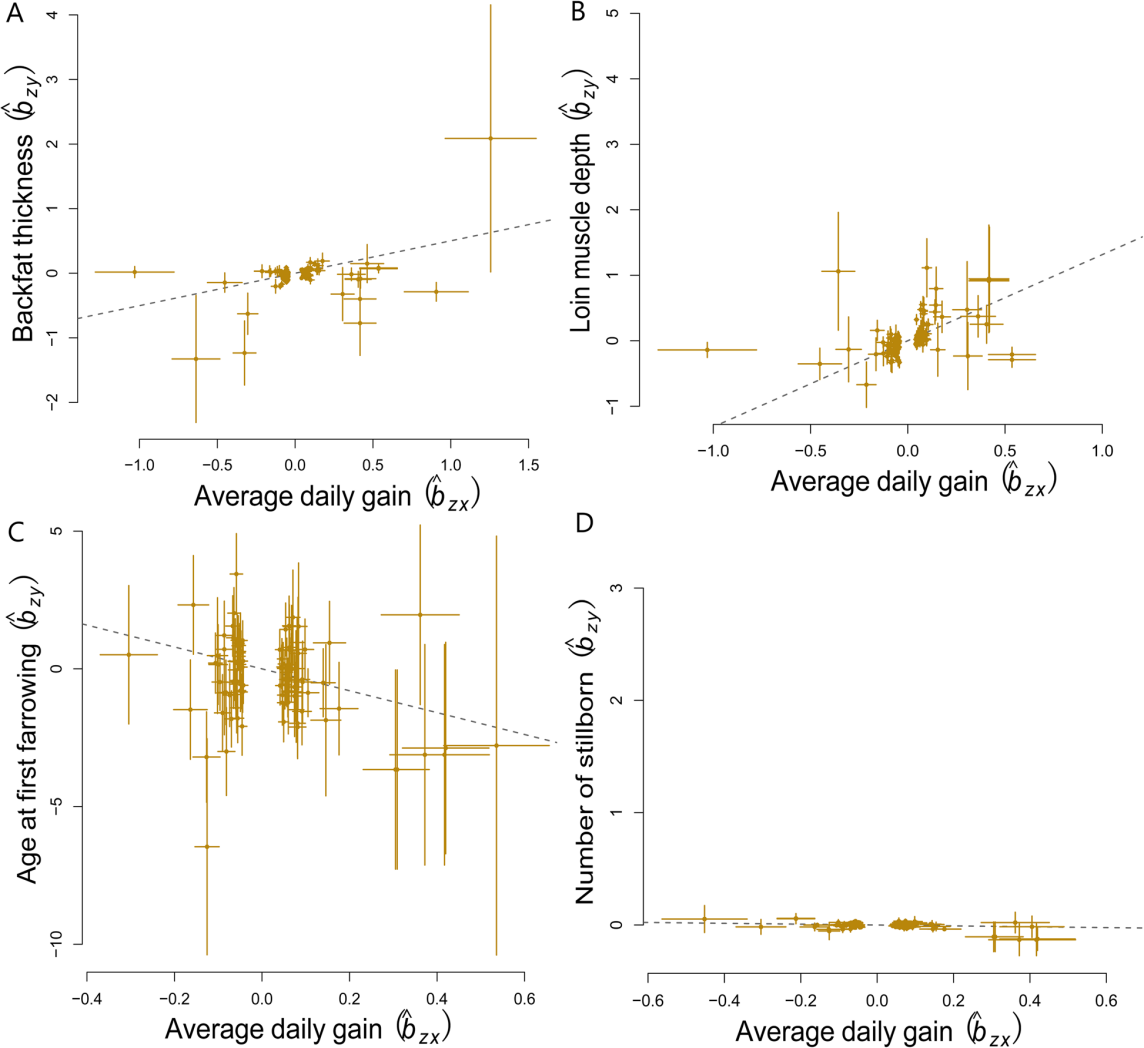
The GSMR analysis of average daily gain on key carcass composition and reproductive traits in pigs. **A** Backfat thickness (BFT), **B** Loin muscle depth (LMDEP), **C** Age at first farrowing (AFF), **D** Number of stillborn (NSB)

Our MR analysis identified associations consistent with potential causal effects of ADG on key meat and reproductive traits in pigs. For carcass composition traits, ADG showed positive effects consistent with a possible causal role on BFT ($\widehat{b}{ }_{xy}$= 0.13 ± 0.04, $P$ < 0.05) and LMDEP ($\widehat{b}{ }_{xy}$= 0.35 ± 0.07, $P$ < 0.05) were positive (Table 3; Fig. 1A and B). For reproductive traits, negative associations indicating a possible causal influence of ADG were observed for AFF ($\widehat{b}{ }_{xy}$= −3.24 ± 1.29, $P$ = 0.0124), while a statistically significant but small negative effect suggests a potential causal link for NSB ($\widehat{b}{ }_{xy}$= −0.05 ± 0.02, $P$ < 0.05) (Table 3; Fig. 1C and D). All associations remained significant after Bonferroni correction for multiple testing (*α* = *0.05*).

**Table 3:** Causal relationships between ADG and key production and reproductive traits in pigs

Traits	${SNP}_{OUT}$	${SNP}_{INDEX}$	$\widehat{b}{ }_{xy} \left(SE\right)$	${P}_{GSMR}$	IVs
BFT	273	119	0.13 (0.04)	2.50 × 10^−^^4^	115
LMDEP	273	115	0.35 (0.07)	2.40 × 10^−^^6^	113
AFF	273	106	−3.24 (1.29)	1.24 × 10^−^^2^	106
NSB	273	112	−0.05 (0.02)	7.20 × 10^−^^3^	112

${SNP}_{OUT}$ represents the number of genome-wide significant (${P}_{GSMR}<5\times 1{0}^{-8}$) SNPs for combined exposure and outcome pairs; ${SNP}_{INDEX}$: The number of SNPs after the additional clumping filter; $\widehat{b}{ }_{xy}$: Estimated effect sizes from the GSMR analysis are presented along with their standard errors (*SE*); ${P}_{GSMR}$: $P$-values of GSMR; IVs: The number of significant SNPs used to infer causality. *ADG* Average daily gain, *AFF *Age at first farrowing, *BFT* Backfat thickness, *LMDEP* Loin muscle depth, *NSB *Number of stillborn

### Candidate gene links ADG to carcass composition and reproductive traits

For each exposure–outcome analysis, we identified trait-specific genes contributing to potential causal inference of exposure on the respective outcome traits (Additional file 1: Table S2). A total of 115 IVs were associated with BFT, corresponding to 47 annotated genes. Likewise, LMDEP analysis revealed 113 IVs linked to 44 genes. For AFF, 106 IVs mapped to 47 genes, whereas 112 IVs related to NSB, resulting in 40 annotated genes. Among the annotated genes across the four exposure–outcome analyses, 59.6% (31 genes) were shared by all four traits, including *MYOM1*, *MYO3B*, *MC4R*, and *CDH20* (Additional file 1: Table S2, Additional file 2: Fig. S1).

### Shared genetic signals for ADG, carcass composition, and reproduction traits

**Fig. 2:**
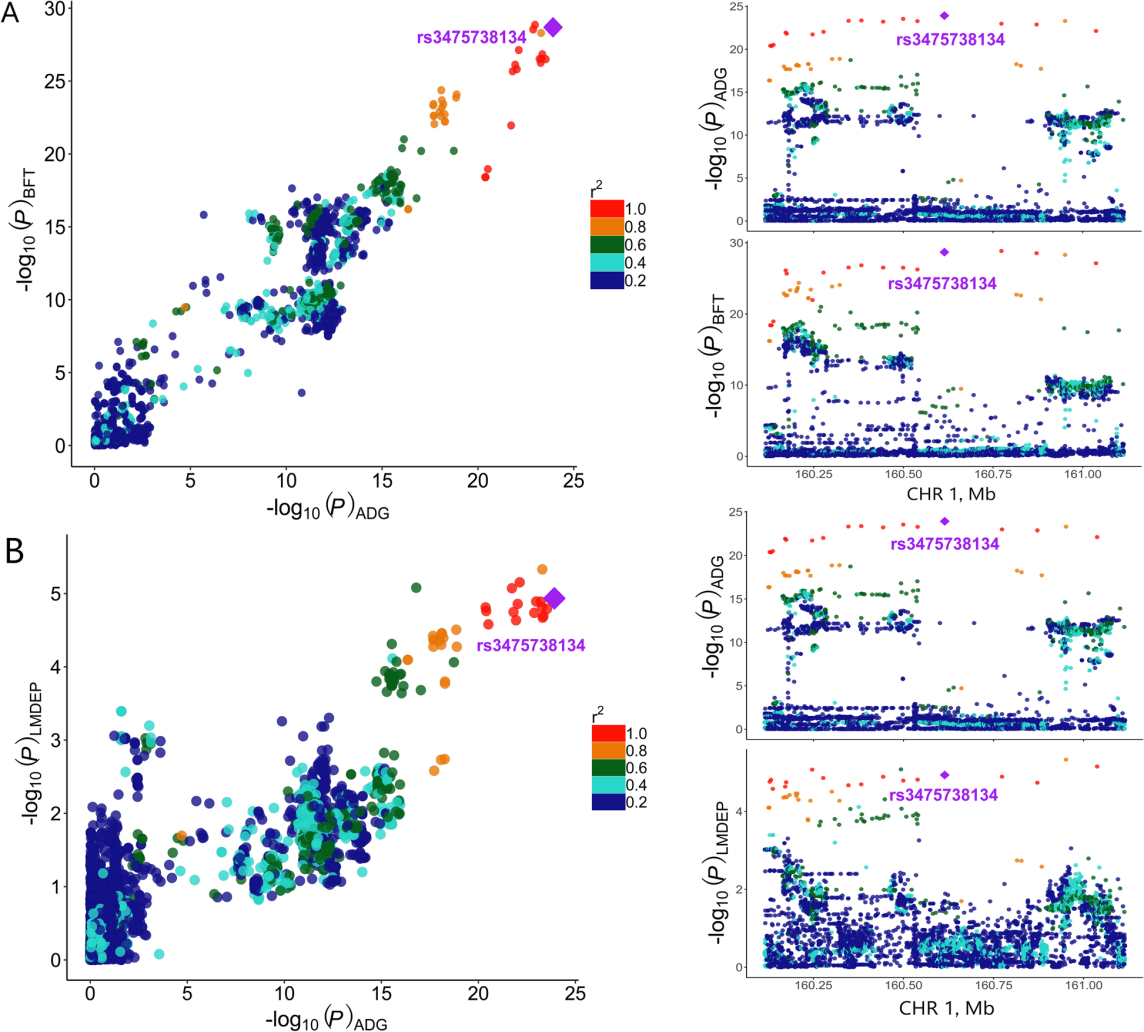
Colocalization of ADG with two carcass composition traits. **A** Local Manhattan plots showing the colocalization between average daily gain (ADG) and backfat thickness (BFT). The left panel displays GWAS signals for ADG and BFT in the shared locus. The right panel highlights ± 500 kb around the top colocalized SNP. **B** Local Manhattan plots showing the colocalization between ADG and lean meat depth (LMDEP). The left panel displays GWAS signals for ADG and LMDEP in the shared locus. The right panel highlights ± 500 kb around the top colocalized SNP. The purple diamond indicates the top colocalized SNP, and the color bar displays LD information

Colocalization analysis provided strong evidence for shared genetic regulation between ADG and BFT, as well as between ADG and LMDEP, with posterior probabilities supporting a shared causal variant exceeding 0.99 ($$PP.H4>0.99)$$ (Additional file 1: Tables S3). The SNP rs3475738134 emerged as the top-associated variant in both trait comparisons (Fig. 2A and B). To further assess the robustness of this signal, we performed additional colocalization analyses using narrower genomic windows (± 100 kb and ± 250 kb). These analyses consistently identified the same top SNP (rs3475738134) with high PP.H4 (> 0.99) in both BFT and LMDEP, confirming a shared causal variant across window sizes (Additional file 3: Fig. S2A–S2D). Similarly, colocalization analysis between ADG and AFF identified the same SNP (rs3475738134) as a top candidate, with strong evidence for shared genetic regulation ($$PP.H4>0.90$$) (Additional file 1: Table S3, Additional file 4: Fig. S3A). In contrast, colocalization analysis between ADG and NSB yielded a *PP.H*4 of 0.49, which likely reflects limited statistical power rather than convincing colocalization (Additional file 1: Table S3; Additional file 4: Fig. S3B). Further analyses using ± 100 kb and ± 250 kb windows produced consistent results for both reproductive traits tested, similar to those observed with the previously tested ± 500 kb window (Additional file 5: Fig. S4A–S4D). To investigate the potential functional relevance of the colocalized regions, annotations of top variants overlapped with 25 genes, including *MC4R* and *CDH20*, which showed significant associations with the traits of interest (ADG, BFT, LMDEP, and AFF) (Additional file 1: Table S4).

**Fig. 3:**
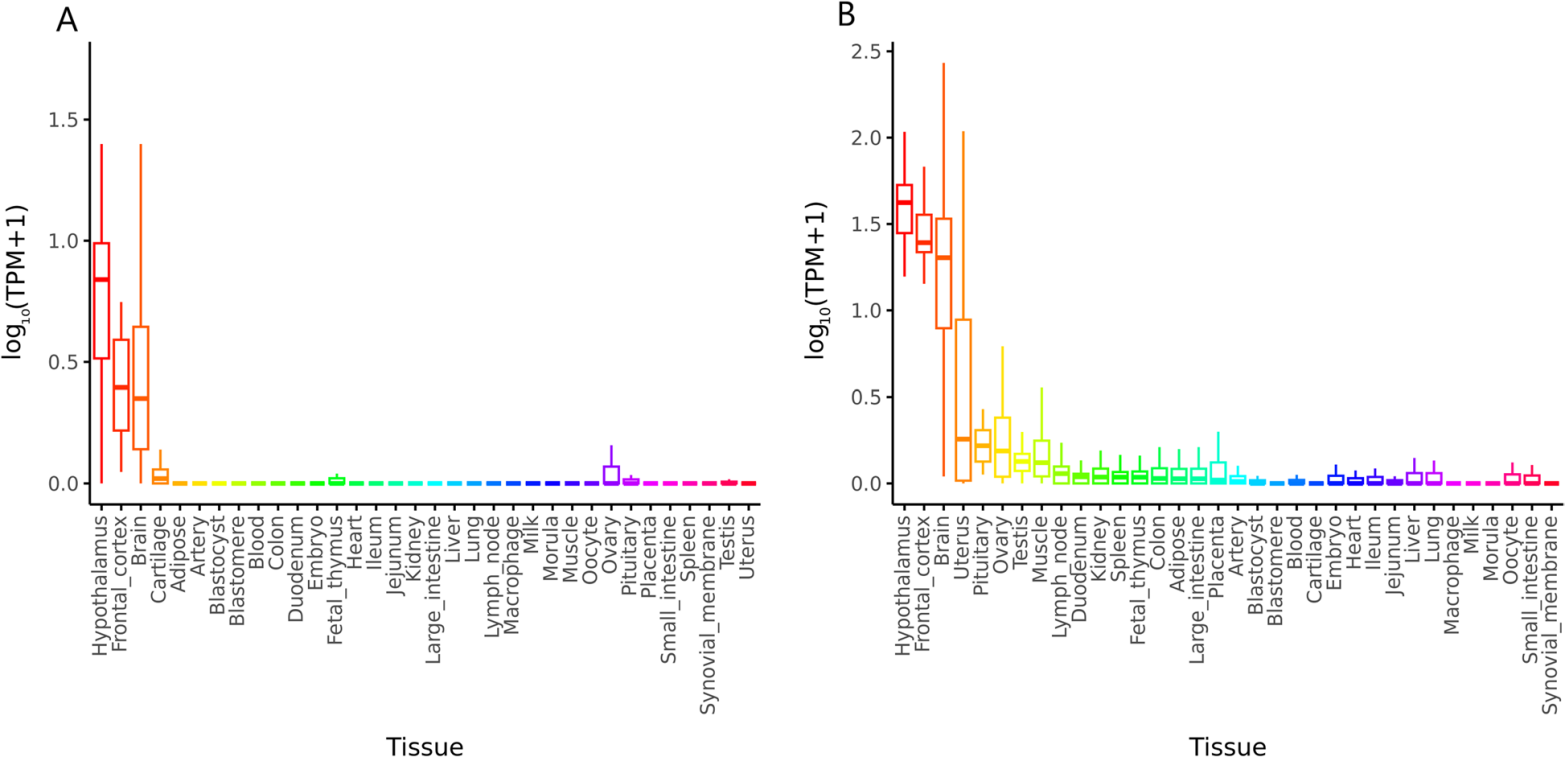
Gene expression levels in 34 different tissues from the PigGTEx project. **A** Gene expression for the *MC4R* gene. **B** Gene expression for the *CDH20 *gene. Both genes exhibit significant tissue-specific differences in expression (*P* < 1 × 10^−^^8^)

**Fig. 4:**
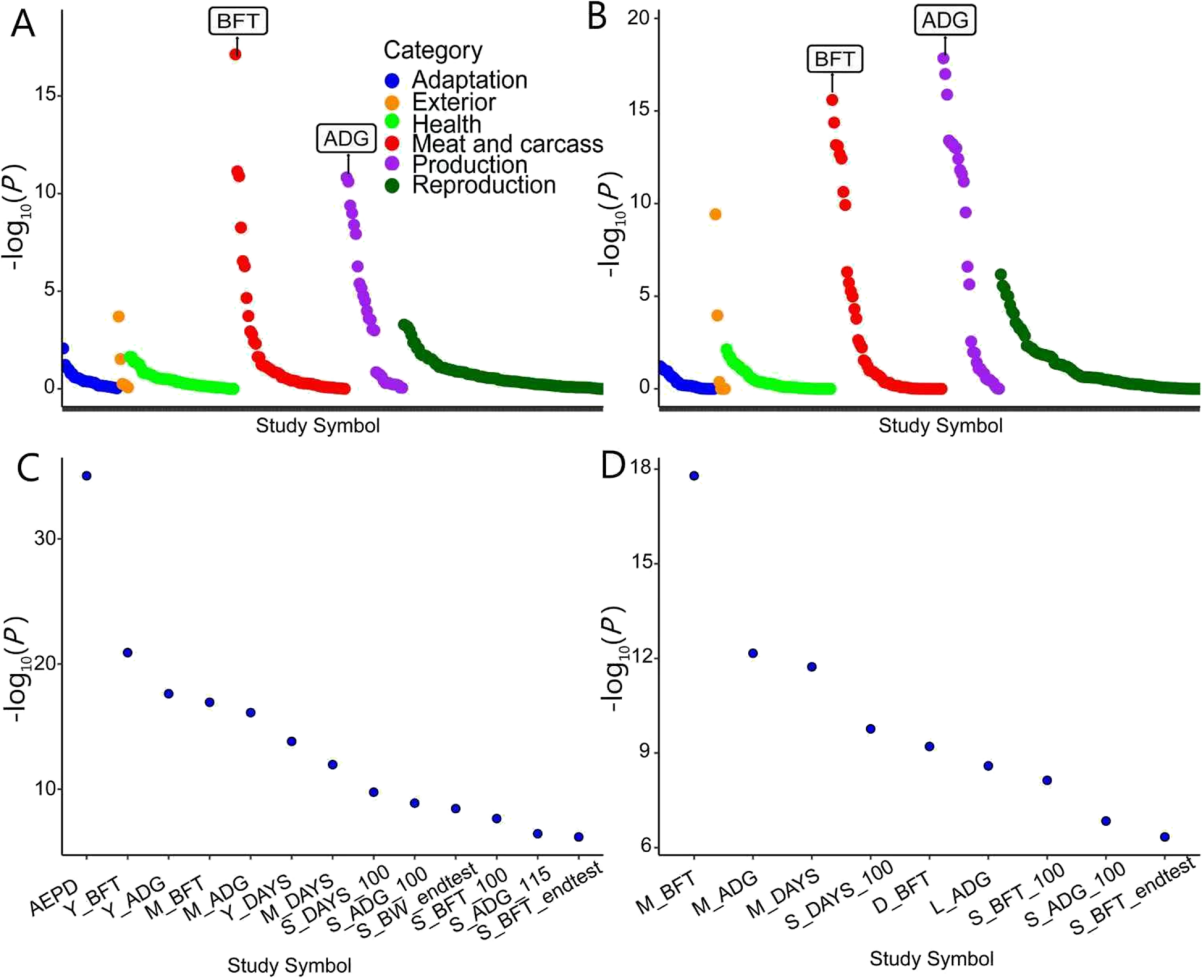
PheWAS and TWAS analyses from PigBiobank. **A** PheWAS for *MC4R* across 298 complex traits. The *x*-axis represents individual traits used in PigBiobank categories; the *y*-axis represents the statistical significance of association (−log_10_(*P*-value)). **B** PheWAS for *CDH20* across the same 298 complex traits. The *x*-axis and *y*-axis are defined as in panel A. **C** TWAS for *MC4R* in muscle and reproductive tissues. The *x*-axis shows different complex traits used in PigBiobank; *y*-axis represents the statistical significance of transcript–trait associations (−log_10_(*P*-value)). **D** TWAS for *CDH20* in the same muscle and reproductive tissues. *x*-axis and *y*-axis follow panel C. Data were accessed from PigBiobank (http://pigbiobank.farmgtex.org)

We further prioritized candidate genes associated with the traits of interest that overlapped between the annotated variants identified in the MR and colocalization analyses. We then investigated their tissue-specific expression profiles across 34 different pig tissues. Gene expression analysis revealed that both *MC4R* and *CDH20* exhibit relatively high expression levels in the hypothalamus, frontal cortex, and other brain regions, with the hypothalamus showing the highest expression for both genes (Fig. 3A and B). Integrative eQTL analysis revealed tissue-specific regulatory effects for these genes. *MC4R* harbored 61 significant *cis-*eQTLs, with the strongest signals observed in the frontal cortex (Additional file 1: Table S5), while *CDH20* showed 17 significant *cis-*eQTLs, predominantly in muscle tissue (Additional file 1: Table S6). LD analysis revealed that the top colocalized SNP (rs3475738134) was moderately correlated (*r*^*2*^ > 0.65) with a nearby *cis-*eQTL SNP for *MC4R* in the frontal cortex (Additional file 1: Table S14). For *CDH20*, significant *cis-*eQTLs were detected in muscle tissue; however, these eQTLs were not in high LD with the top colocalized SNP (Additional file 1: Table S7). PheWAS across 298 traits further highlighted that *MC4R* and *CDH20* are significantly associated with BFT and ADG (Fig. 4A and B). Moreover, TWAS results from 34 tissues confirmed that *MC4R* is significantly associated with ADG and BFT in the frontal cortex (Fig. 4C). For *CDH20*, the significant TWAS results revealed a link between this gene and ADG and BFT in muscle tissue (Fig. 4D).

**Fig. 5:**
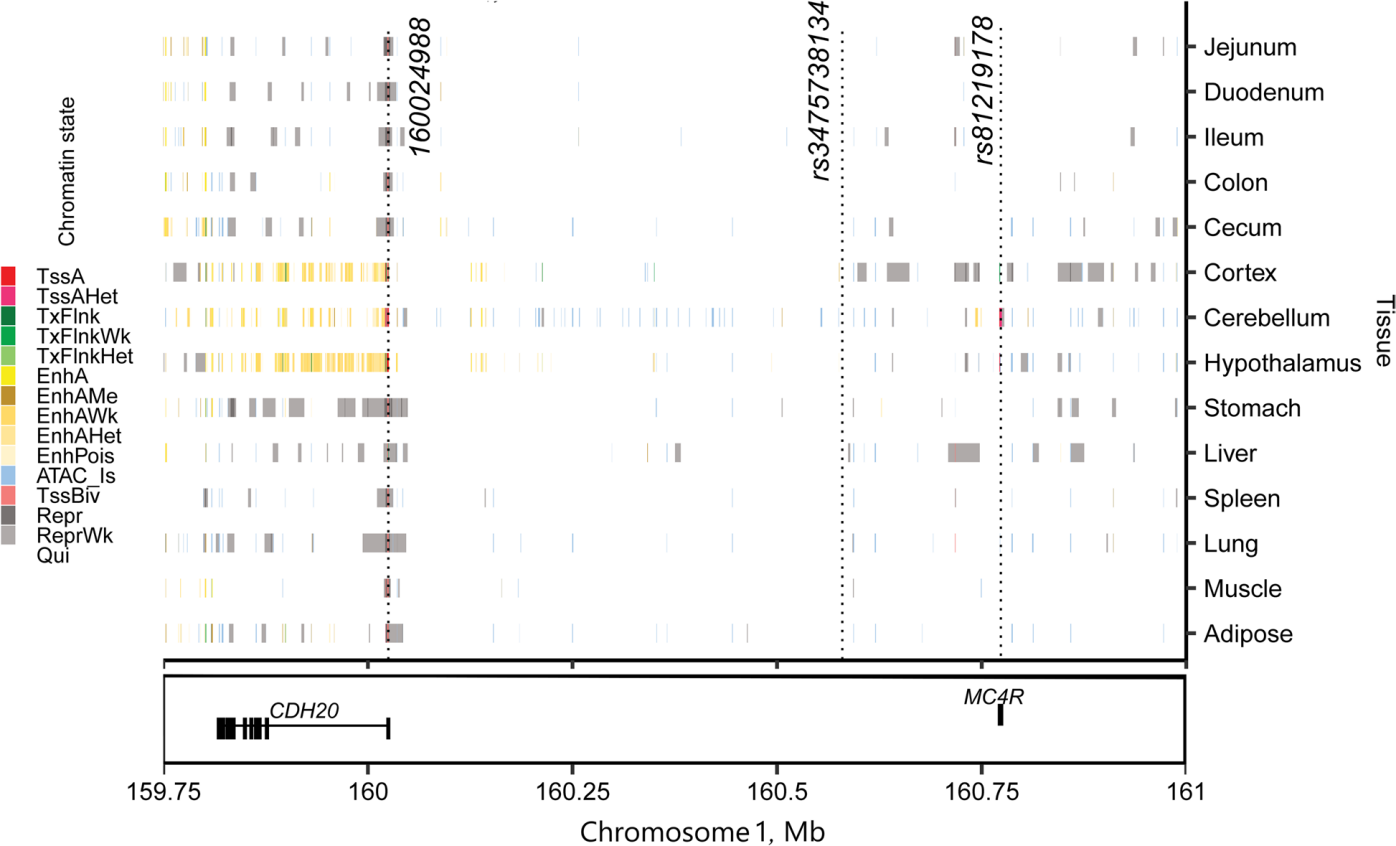
Tissue- and chromatin-state-specific annotation of the *CDH20* and *MC4R* locus across 14 pig tissues. Each row represents a chromatin state (left) or tissue type (right). Colors correspond to specific chromatin states. TssA: Strongly active promoters/transcripts; TssAHet: Flanking active TSS without ATAC; TxFlnk: Transcribed at gene; TxFlnkWk: Weak transcribed at gene; TxFlnkHet: Transcribed region without ATAC; EnhA: Strong active enhancer; EnhAMe: Medium enhancer with ATAC; EnhAWk: Weak active enhancer; EnhAHet: Active enhancer no ATAC (hetero); EnhPois: Poised enhancer; ATAC_Is: ATAC island; TssBiv: Bivalent/poised TSS; Repr: Repressed polycomb; ReprWk: Weak repressed polycomb; Qui: Quiescent. Vertical dashed lines indicate the positions of three variants of interest: a high-LD SNP near *CDH20* (position 160,024,988), the *MC4R* coding variant (rs8129178), and the top colocalized SNP (rs3475738134). This figure integrates epigenomic information to visualize regulatory potential and tissue-specific chromatin accessibility in the region

Following strong evidence for shared genetic regulation from colocalization analysis, we further characterized this region to investigate functional features. VEP annotations (Additional file 1: Table S8) of the top colocalized SNP (rs3475738134) and its high LD proxies (*r*^2^ ≥ 0.8) revealed key functional insights in the *MC4R* gene. The top colocalized SNP map is located upstream of the lncRNA ENSSSCG00000060219, while a high LD SNP (rs81219178) within *MC4R* is a missense variant (*Asp298Asn*). Additionally, another high LD SNP, located at position 160,024,988, is situated upstream of the lncRNA ENSSSCG00000061175, near the *CDH20* gene. Figure 5 suggests regulatory activity at these loci, as indicated by chromatin state annotations. Specifically, for the *MC4R* locus, which harbors the missense SNP rs81219178, chromatin state signals in brain tissues (cortex, cerebellum, and hypothalamus) overlapped with strongly active promoters/transcripts (TssA) and Flanking active TSS without ATAC (TssAHet), supporting regulatory potential in neural contexts. At the *CDH20* locus, chromatin accessibility signals corresponding to TssAHet were predominantly detected in muscle and adipose tissues, suggesting regulatory activity that may contribute to growth and fat deposition.

## Discussion

This study combined MR and colocalization analysis to estimate the causal effects of ADG on carcass composition and reproductive traits in pigs. A key challenge in MR analyses is the potential for horizontal pleiotropy to bias causal estimates [13]. To address this, we implemented the HEIDI-outlier test [30], which identified and excluded six horizontal pleiotropic variants, enhancing the robustness of our causal inference. Notably, three excluded variants were located near genes with established roles in growth and carcass composition traits: a SNP at SSC1:158,058,352 lies approximately 500 kb downstream of *PHLPP1*, previously implicated in ADG and BFT traits in pigs [40, 41]; this same SNP is also ~ 175 kb downstream of *VPS4B*, associated with ADG and BFT in pigs [41], and the same gene with ADG in Simmental and Simbrah cattle [42]. Additionally, pleiotropic signals near *COL21A1*—located approximately 283 kb upstream of SSC7:29,415,089–29,609,036, a region associated with ADG and BFT [43] and involved in muscle growth regulation [44, 45].

After accounting for horizontal pleiotropy, MR identified a potential causal effect of ADG on both BFT and LMDEP, consistent with the biological expectation that faster-growing pigs have increased energy intake leading to greater tissue accretion, including both fat and muscle [46, 47]. Feed intake measured early in growth shows stronger genetic correlations with leanness than later intake [48], suggesting that feed intake may serve as an intermediate phenotype linking ADG to these carcass composition traits. This reflects mediated pleiotropy, in which genetic variants affect the exposure (ADG), and the effect of ADG subsequently mediates the influence on the outcome traits [12, 49]. Interestingly, we observed a negative causal effect of ADG on AFF, indicating that faster growth accelerates reproductive maturity. This aligns with negative genetic correlations between growth rate and AFF in pigs [28, 50] and with the association between fast growth and earlier first calving in Nellore cattle [51]. Faster growth is likely to promote earlier onset of puberty and first estrus [52, 53], highlighting the co-regulation of growth and reproductive pathways through shared metabolic and hormonal signals. Our analysis also identified a negative causal effect of ADG on NSB, with a modest effect size (Table 3). Although this finding suggests a potential genetic link between faster growth and reduced stillbirth risk, the result warrants cautious interpretation and further validation.

In addition to the statistical evidence, gene annotation of IVs revealed a set of overlapping candidate genes with potential physiological relevance, which may help explain the causal associations observed across the four exposure–outcome analyses. Among the 40 overlapping genes (Additional file 1: Table S6), *MYOM1*, *MYO3B*, *MC4R*, and *CDH20* highlight potential shared loci involved in muscle development, energy balance, and tissue remodeling. Notably, polymorphisms in *MYOM1* have been shown to increase loin weight, primary cut weight, and backfat without reducing meat quality [54], supporting the putative causal effects of ADG on carcass composition traits. Similarly, *MYO3B* variants are associated with skin-on whole loin [55], a trait reflecting muscle area and overall carcass composition, providing further biological prospect for ADG’s causal impact on carcass composition traits.

Building on our MR findings, the subsequent colocalization analysis provides additional evidence of coordinated genetic regulation, with consistent support, at the *MC4R* and *CDH20* genes, illustrating shared genetic influences across growth, carcass composition, and reproductive traits. The well-characterized *MC4R Asp298Asn* variant is consistently associated with increased ADG and BFT alongside reduced carcass leanness [56–58]. Our MR results align, showing that faster-growing pigs accumulate both fat and muscle (BFT and LMDEP); however, greater fat deposition relative to muscle likely results in a lower carcass lean proportion. Integrative evidence from chromatin accessibility, *cis-*eQTLs, TWAS, and PheWAS highlights the central role of *MC4R* in growth and energy balance. Its predominant expression in the hypothalamus and frontal cortex, key centers for appetite and metabolic regulation, further supports its involvement in growth and fat deposition [56, 59, 60]. The colocalization of ADG and AFF at *MC4R* reflects pleiotropic effects that coordinate growth and reproductive timing, consistent with its metabolic regulatory functions [61, 62], positioning *MC4R* as a nexus linking energy balance with productivity and reproduction. Similarly, *CDH20* is predominantly expressed in muscle tissue, where it contributes to myogenesis and cytoskeletal integrity [63], as well as lipid accumulation and muscle remodeling [41]. Our TWAS and PheWAS analyses support its pleiotropic influence on growth and adiposity traits. Although no direct *cis-*eQTL was detected, regulatory mechanisms beyond local expression control may underlie *CDH20’*s function, warranting further investigation.

Together, these findings reveal shared genetic architecture and potential causal relationships among growth, carcass composition, and reproductive traits in pigs, advancing our understanding of their biological interplay. Particularly, causal studies leveraging large-scale GWAS and multi-omics integration are essential, as moving beyond association toward mechanistic understanding supports the development of multi-trait breeding strategies that optimize productivity while managing biological trade-offs. We acknowledge that using GWAS summary statistics from the same population for both the exposure and outcome may introduce a risk of confounding. However, fully independent datasets are rarely available in animal genetics, representing a common limitation in the field [12]. Additionally, due to a limited number of independent IVs after quality control, we were unable to perform reverse MR analyses to assess bidirectional causal relationships. However, by integrating gene annotation, colocalization, and functional genomics resources, we strengthened the biological plausibility of our findings.

## Conclusions

This study used MR and colocalization analysis to explore potential causal links between growth, carcass composition, and reproductive traits in pigs. Our results provide genetic evidence for a potential causal relationship between growth traits and both fat deposition and reproductive timing. By integrating MR and colocalization results with functional genomic resources from PigGTEx, we identified candidate genes, such as *MC4R* and *CDH20, *that may contribute to the coordinated regulation of growth, fat deposition, and reproductive timing. These findings contribute to genetic links between production and reproductive traits. As both exposure and outcome statistics were derived from the same meta-GWAS, the possibility of residual confounding cannot be entirely excluded. Therefore, independent replication in larger datasets will be essential to validate these findings and assess potential reverse causality.

## Supplementary Information

Additional file 1: Table S1. Description of the meta-GWAS summary statistics used in the GSMR-based Mendelian randomization and colocalization analyses. Table S2. Summary of candidate genes associated with carcass composition and reproductive traits, directly annotated to GSMR instrumental SNPs. Table S3. Summary of colocalization analysis results and numbers of SNPs used across carcass composition and reproductive traits. Table S4. Genomic coordinates and annotations of genes in the top colocalized region. Table S5. Significant *cis*-eQTLs associated with MC4R expression in pig frontal cortex. Table S6. Significant *cis*-eQTLs associated with *CDH20* expression in pig muscle tissue. Table S7. LD between the top colocalized SNPand *cis*-eQTL SNPs in the *MC4R* and *CDH20* genes. Table S8. Functional annotation of top colocalized and high-LD SNPs at the *MC4R* and *CDH20* loci, including genomic positions, predicted variant consequences, and associated traits from QTLdb.

Additional file 2: Fig. S1. Venn diagram of shared candidate genes identified through MR analyses between ADG and carcass composition traitsand reproductive traits.

Additional file 3: Fig. S2. Local Manhattan plots illustrate the colocalization between ADG and carcass composition traits using different genomic window sizes. A Colocalization between ADG and BFT within a ±100 kb window centered on the top colocalized SNP. B Colocalization between ADG and BFT within a ±250 kb window. C Colocalization between ADG and LMDEP within a ±100 kb window. D Colocalization between ADG and LMDEP within a ±250 kb window. For each panel, the left plot displays GWAS association signals for ADG and the corresponding carcass trait across the shared locus, while the right plot zooms into the specified window centered on the top colocalized SNP. SNPs are colored according to LD with the top colocalized SNP.

Additional file 4: Fig. S3. Colocalization of ADG with reproductive traits within a ±500 kb window. A Local Manhattan plots showing the colocalization between ADG and AFF. B Local Manhattan plots showing the colocalization between ADG and NSB. For each panel, the left plot displays GWAS association signals for ADG and the corresponding reproductive trait across the shared locus, while the right plot zooms into a ±500 kb window centered on the top colocalized SNP. SNP colors represent LD with the top colocalized SNP.

Additional file 5: Fig. S4. Local Manhattan plots illustrate the colocalization between ADG and reproductive traits using different genomic window sizes. A Colocalization between ADG and AFF within a ±100 kb window around the lead colocalized SNP. B Colocalization between ADG and AFF within a ±250 kb window. C Colocalization between ADG and NSB within a ±100 kb window. D Colocalization between ADG and NSB within a ±250 kb window. For each panel, the left plot shows the GWAS association signals for ADG and the corresponding reproductive trait across the locus, while the right plot zooms into the specified window centered on the top colocalized SNP. The purple diamond denotes the top colocalized SNP, and the color scale indicates LD relative to this SNP.

## Data Availability

The meta-GWAS summary statistics used for this study can be made available from the corresponding author upon reasonable request.

## Abbreviations

ADG: Average daily gain
AFF: Age at first farrowing
BFT: Backfat thickness
*cis-*eQTLs: *Cis*-expression quantitative trait loci
eQTL: Expression quantitative trait loci
FarmGTEx: Farm Animal Genotype–Tissue Expression
GSMR: Generalised summary-data-based Mendelian randomisation
GWAS: Genome-wide association studies
HEIDI: Heterogeneity in dependent instruments
IVs: Instrumental variants
LD: Linkage disequilibrium
LMDEP: Loin muscle depth
MR: Mendelian randomization
NSB: Number of stillborn piglets
PGRP v1: Pig Genomics Reference Panel version 1
PheWAS: Phenome-wide association studies
PigGTEx: Pig Genotype-Tissue Expression
RCTs: Randomized controlled trials
SSC: Sus scrofa chromosomes
TWAS: Transcriptome-wide association studies

## References

[1] Meuwissen TH, Hayes BJ, Goddard M. Prediction of total genetic value using genome-wide dense marker maps. Genetics. 2001;157(4):1819–29. 10.1093/genetics/157.4.1819. 11290733 10.1093/genetics/157.4.1819 PMC1461589

[2] Hayes BJ, Bowman PJ, Chamberlain AJ, Goddard ME. Invited review: Genomic selection in dairy cattle: progress and challenges. J Dairy Sci. 2009;92(2):433–43. 10.3168/jds.2008-1646. 19164653 10.3168/jds.2008-1646

[3] Ducos A, Bidanel JP. Genetic correlations between production and reproductive traits measured on the farm, in the Large White and French Landrace pig breeds. J Anim Breed Genet. 1996;113(1–6):493–504. 10.1111/j.1439-0388.1996.tb00639.x.

[4] Holm B, Bakken M, Klemetsdal G, Vangen O. Genetic correlations between reproduction and production traits in swine. J Anim Sci. 2004;82(12):3458–64. 10.2527/2004.82123458x. 15537764 10.2527/2004.82123458x

[5] Kanis E, De Greef KH, Hiemstra A, Van Arendonk JA. Breeding for societally important traits in pigs. J Anim Sci. 2005;83(4):948–57. 10.2527/2005.834948x. 15753352 10.2527/2005.834948x

[6] Alam M, Chang HK, Lee SS, Choi TJ. Genetic analysis of major production and reproduction traits of Korean Duroc, Landrace and Yorkshire pigs. Animals. 2021;11(5):1321. 10.3390/ani11051321. 34063090 10.3390/ani11051321 PMC8147943

[7] Schwarz L, Heise J, Liu Z, Bennewitz J, Thaller G, Tetens J. Mendelian randomisation to uncover causal associations between conformation, metabolism, and production as potential exposure to reproduction in German Holstein dairy cattle. Genet Sel Evol. 2025;57:7. 10.1186/s12711-025-00950-w. 40000939 10.1186/s12711-025-00950-w PMC11863791

[8] Wei R, Zhang Z, Han H, Miao J, Yu P, Cheng H, et al. Integrative genomic analysis reveals shared loci for reproduction and production traits in Yorkshire pigs. BMC Genomics. 2025;26:310. 10.1186/s12864-025-11416-0. 40158163 10.1186/s12864-025-11416-0 PMC11954345

[9] Xu Z, Lin Q, Cai X, Zhong Z, Teng J, Li B, et al. Integrating large-scale meta-GWAS and PigGTEx resources to decipher the genetic basis of 232 complex traits in pigs. Natl Sci Rev. 2025;12(5):nwaf048. 10.1093/nsr/nwaf048. 40330097 10.1093/nsr/nwaf048 PMC12051865

[10] Cartwright N. What are randomized controlled trials good for? Philos Stud. 2010;147(1):59–70. 10.1007/s11098-009-9450-2.

[11] Pickrell JK. Fulfilling the promise of Mendelian randomization. bioRxiv. 2015. 10.1101/018150.

[12] Schneider H, Haas V, Krizanac AM, Falker-Gieske C, Heise J, Tetens J, et al. Mendelian randomization analysis of 34,497 German Holstein cows to infer causal associations between milk production and health traits. Genet Sel Evol. 2024;56:27. 10.1186/s12711-024-00896-5. 38589805 10.1186/s12711-024-00896-5 PMC11000328

[13] Burgess S, Butterworth A, Thompson SG. Mendelian randomization analysis with multiple genetic variants using summarized data. Genet Epidemiol. 2013;37(7):658–65. 10.1002/gepi.21758. 24114802 10.1002/gepi.21758 PMC4377079

[14] Davey Smith G, Hemani G. Mendelian randomization: genetic anchors for causal inference in epidemiological studies. Hum Mol Genet. 2014;23(R1):R89-98. 10.1093/hmg/ddu328. 25064373 10.1093/hmg/ddu328 PMC4170722

[15] Yuan S, Chen J, Ruan X, Sun Y, Zhang K, Wang X, et al. Smoking, alcohol consumption, and 24 gastrointestinal diseases: Mendelian randomization analysis. Elife. 2023;12:e84051. 10.7554/eLife.84051. 36727839 10.7554/eLife.84051 PMC10017103

[16] Giambartolomei C, Vukcevic D, Schadt EE, Franke L, Hingorani AD, Wallace C, et al. Bayesian test for colocalisation between pairs of genetic association studies using summary statistics. PLoS Genet. 2014;10(5):e1004383. 10.1371/journal.pgen.1004383. 24830394 10.1371/journal.pgen.1004383 PMC4022491

[17] Zuber V, Grinberg NF, Gill D, Manipur I, Slob EA, Patel A, et al. Combining evidence from Mendelian randomization and colocalization: review and comparison of approaches. Am J Hum Genet. 2022;109(5):767–82. 10.1016/j.ajhg.2022.04.001. 35452592 10.1016/j.ajhg.2022.04.001 PMC7612737

[18] Zheng J, Haberland V, Baird D, Walker V, Haycock PC, Hurle MR, et al. Phenome-wide Mendelian randomization mapping the influence of the plasma proteome on complex diseases. Nat Genet. 2020;52(10):1122–31. 10.1038/s41588-020-0682-6. 32895551 10.1038/s41588-020-0682-6 PMC7610464

[19] Albert FW, Kruglyak L. The role of regulatory variation in complex traits and disease. Nat Rev Genet. 2015;16(4):197–212. 10.1038/nrg3891. 25707927 10.1038/nrg3891

[20] Frydas A, Wauters E, van der Zee J, Van Broeckhoven C. Uncovering the impact of noncoding variants in neurodegenerative brain diseases. Trends Genet. 2022;38(3):258–72. 10.1016/j.tig.2021.08.010. 34535299 10.1016/j.tig.2021.08.010

[21] Fang L, Teng J, Lin Q, Bai Z, Liu S, Guan D, et al. The Farm Animal Genotype–Tissue Expression (FarmGtex) project. Nat Genet. 2025. 10.1038/s41588-025-02121-5. 10.1038/s41588-025-02121-5 40097783

[22] Teng J, Gao Y, Yin H, Bai Z, Liu S, Zeng H, et al. A compendium of genetic regulatory effects across pig tissues. Nat Genet. 2024;56(1):112–23. 10.1038/s41588-023-01585-7. 38177344 10.1038/s41588-023-01585-7 PMC10786720

[23] Zeng H, Zhang W, Lin Q, Gao Y, Teng J, Xu Z, et al. PigBiobank: a valuable resource for understanding genetic and biological mechanisms of diverse complex traits in pigs. Nucleic Acids Res. 2024;52(D1):D980–9. 10.1093/nar/gkad1080. 37956339 10.1093/nar/gkad1080 PMC10767803

[24] Browning BL, Zhou Y, Browning SR. A one-penny imputed genome from next-generation reference panels. Am J Hum Genet. 2018;103(3):338–48. 10.1016/j.ajhg.2018.07.015. 30100085 10.1016/j.ajhg.2018.07.015 PMC6128308

[25] Jiang L, Zheng Z, Qi T, Kemper KE, Wray NR, Visscher PM, et al. A resource-efficient tool for mixed model association analysis of large-scale data. Nat Genet. 2019;51(12):1749–55. 10.1038/s41588-019-0530-8. 31768069 10.1038/s41588-019-0530-8

[26] Winkler TW, Day FR, Croteau-Chonka DC, Wood AR, Locke AE, Mägi R, et al. Quality control and conduct of genome-wide association meta-analyses. Nat Protoc. 2014;9(5):1192–212. 10.1038/nprot.2014.071. 24762786 10.1038/nprot.2014.071 PMC4083217

[27] Willer CJ, Li Y, Abecasis GR. METAL: fast and efficient meta-analysis of genomewide association scans. Bioinformatics. 2010;26(17):2190–1. 10.1093/bioinformatics/btq340. 20616382 10.1093/bioinformatics/btq340 PMC2922887

[28] Serenius T, Stalder KJ. Genetics of length of productive life and lifetime prolificacy in the Finnish Landrace and Large White pig populations. J Anim Sci. 2004;82(11):3111–7. 10.2527/2004.82113111x. 15542456 10.2527/2004.82113111x

[29] Serenius T, Sevón-Aimonen ML, Kause A, Mantysaari EA, Maki-Tanila A. Genetic associations of prolificacy with performance, carcass, meat quality, and leg conformation traits in the Finnish Landrace and Large White pig populations. J Anim Sci. 2004;82(8):2301–6. 10.2527/2004.8282301x. 15318728 10.2527/2004.8282301x

[30] Zhu Z, Zheng Z, Zhang F, Wu Y, Trzaskowski M, Maier R, et al. Causal associations between risk factors and common diseases inferred from GWAS summary data. Nat Commun. 2018;9:224. 10.1038/s41467-017-02317-2. 29335400 10.1038/s41467-017-02317-2 PMC5768719

[31] Xue A, Zhu Z, Wang H, Jiang L, Visscher PM, Zeng J, et al. Unravelling the complex causal effects of substance use behaviours on common diseases. Commun Med. 2024;4:43. 10.1038/s43856-024-00473-3. 38472333 10.1038/s43856-024-00473-3 PMC10933313

[32] Yang J, Lee SH, Goddard ME, Visscher PM. GCTA: a tool for genome-wide complex trait analysis. Am J Hum Genet. 2011;88(1):76–82. 10.1016/j.ajhg.2010.11.011. 21167468 10.1016/j.ajhg.2010.11.011 PMC3014363

[33] Revez JA, Lin T, Qiao Z, Xue A, Holtz Y, Zhu Z. Genome-wide association study identifies 143 loci associated with 25 hydroxyvitamin D concentration. Nat Commun. 2020;11:1647. 10.1038/s41467-020-15421-7. 32242144 10.1038/s41467-020-15421-7 PMC7118120

[34] McLaren W, Gil L, Hunt SE, Riat HS, Ritchie GR, Thormann A, et al. The ensembl variant effect predictor. Genome Biol. 2016;17:122. 10.1186/s13059-016-0974-4. 27268795 10.1186/s13059-016-0974-4 PMC4893825

[35] Yates AD, Achuthan P, Akanni W, Allen J, Allen J, Alvarez-Jarreta J, et al. Ensembl 2020. Nucleic Acids Res. 2020;48(D1):D682–8. 10.1093/nar/gkz966. 31691826 10.1093/nar/gkz966 PMC7145704

[36] Tweedie S, Braschi B, Gray K, Jones TE, Seal RL, Yates B, et al. Genenames. org: the HGNC and VGNC resources in 2021. Nucleic Acids Res. 2021;49(D1):D939–46. 10.1093/nar/gkaa980.10.1093/nar/gkaa980 PMC7779007 33152070

[37] Wickham H. ggplot2: elegant graphics for data analysis. Springer-Verlag New York. 2016. https://ggplot2.tidyverse.org.

[38] Chen H.VennDiagram: generate high-resolution venn and euler plots: R package version 1.7.3. 2022. https://CRAN.R-project.org/package=VennDiagram. Accessed 7 Oct 2025.

[39] Pan Z, Yao Y, Yin H, Cai Z, Wang Y, Bai L, et al. Pig genome functional annotation enhances the biological interpretation of complex traits and human disease. Nat Commun. 2021;12:5848. 10.1038/s41467-021-26153-7. 34615879 10.1038/s41467-021-26153-7 PMC8494738

[40] Guo Y, Qiu H, Xiao S, Wu Z, Yang M, Yang J, et al. A genome-wide association study identifies genomic loci associated with backfat thickness, carcass weight, and body weight in two commercial pig populations. J Appl Genet. 2017;58(4):499–508. 10.1007/s13353-017-0405-6. 28890999 10.1007/s13353-017-0405-6

[41] Zhang Z, Chen Z, Diao S, Ye S, Wang J, Gao N. Identifying the complex genetic architecture of growth and fatness traits in a Duroc pig population. J Integr Agric. 2021;6:1607–14. 10.1016/S2095-3119(20)63264-6.

[42] Calderón-Chagoya R, Vega-Murillo VE, García-Ruiz A, Ríos-Utrera Á, Martínez-Velázquez G, Montaño-Bermúdez M. Genome and chromosome wide association studies for growth traits in Simmental and Simbrah cattle. Anim Biosci. 2022;36(1):19. 10.5713/ab.21.0517. 35798032 10.5713/ab.21.0517 PMC9834659

[43] Blaj I, Tetens J, Bennewitz J, Thaller G, Falker-Gieske C. Structural variants and tandem repeats in the founder individuals of four F2 pig crosses and implications to F2 GWAS results. BMC Genomics. 2022;23:631. 10.1186/s12864-022-08716-0. 36057580 10.1186/s12864-022-08716-0 PMC9440560

[44] Ghosh M, Sharma N, Gera M, Kim N, Sodhi SS, Pulicherla K, et al. The first comprehensive description of the expression profile of genes involved in differential body growth and the immune system of the Jeju Native Pig and miniature pig. Amino Acids. 2019;51(3):495–511. 10.1007/s00726-018-2685-5. 30519757 10.1007/s00726-018-2685-5

[45] Park J, Lee J, Song KD, Kim SJ, Kim DC, Lee SC, et al. Growth factors improve the proliferation of Jeju black pig muscle cells by regulating myogenic differentiation 1 and growth-related genes. Anim Biosci. 2021;34(8):1392. 10.5713/ab.20.0585. 33561926 10.5713/ab.20.0585 PMC8255883

[46] Hermesch S, Luxford BG, Graser HU. Genetic parameters for lean meat yield, meat quality, reproduction and feed efficiency traits for Australian pigs: 2. Genetic relationships between production, carcase and meat quality traits. Livest Prod Sci. 2000;65(3):249–59. 10.1016/S0301-6226(00)00151-2.

[47] Latorre MA, Pomar C, Faucitano L, Gariépy C, Méthot S. The relationship within and between production performance and meat quality characteristics in pigs from three different genetic lines. Livest Sci. 2008;115(2–3):258–67. 10.1016/j.livsci.2007.08.013.

[48] Von Felde A, Roehe R, Looft H, Kalm E. Genetic association between feed intake and feed intake behaviour at different stages of growth of group-housed boars. Livest Prod Sci. 1996;47(1):11–22. 10.1016/S0301-6226(96)01006-8.

[49] Davies NM, Holmes MV, Smith GD. Reading Mendelian randomisation studies: a guide, glossary, and checklist for clinicians. BMJ. 2018. 10.1136/bmj.k601. 10.1136/bmj.k601 PMC6041728 30002074

[50] Knauer MT, Cassady JP, Newcom DW, See MT. Phenotypic and genetic correlations between gilt estrus, puberty, growth, composition, and structural conformation traits with first-litter reproductive measures. J Anim Sci. 2011;89(4):935–42. 10.2527/jas.2009-2673. 21148777 10.2527/jas.2009-2673

[51] Boligon AA, Albuquerque LG, Mercadante ME, Lôbo RB. Study of relations among age at first calving, average weight gains and weights from weaning to maturity in Nellore cattle. Rev Bras Zootec. 2010;39:746–51. 10.1590/S1516-35982010000400007.

[52] Kummer R, Bernardi ML, Schenkel AC, Amaral Filha WS, Wentz I, Bortolozzo FP. Reproductive performance of gilts with similar age but with different growth rate at the onset of puberty stimulation. Reprod Domest Anim. 2009;44(2):255–9. 10.1111/j.1439-0531.2007.01050.x. 18694424 10.1111/j.1439-0531.2007.01050.x

[53] Magnabosco D, Cunha EC, Bernardi ML, Wentz I, Bortolozzo FP. Effects of age and growth rate at onset of boar exposure on oestrus manifestation and first farrowing performance of Landrace× large white gilts. Livest Sci. 2014;169:180–4. 10.1016/j.livsci.2014.09.013.

[54] Ropka-Molik K, Piórkowska K, Piestrzyńska-Kajtoch A, Fornal A, Zyskowska Z, Pruś A, et al. The poTenTial role of MYOM1 and ATGL genes in pig producTion improvement. Ann Anim Sci. 2021;21(3):833–42. 10.2478/aoas-2020-0102.

[55] Qiu Y, Zhuang Z, Meng F, Ruan D, Xu C, Ma F, et al. Identification of candidate genes associated with carcass component weights in commercial crossbred pigs through a combined GWAS approach. J Anim Sci. 2023;101:skad121. 10.1093/jas/skad121. 37098184 10.1093/jas/skad121 PMC10195203

[56] Klimenko A, Usatov A, Getmantseva L, Kolosov Y, Tretyakova O, Bakoev S, et al. Effects of melanocortin-4 receptor gene on growth and meat traits in pigs raised in Russia. Am J Agri Biol Sci 2014;232–237. 10.3844/ajabssp.2014.232.237.

[57] Ding R, Zhuang Z, Qiu Y, Ruan D, Wu J, Ye J, et al. Identify known and novel candidate genes associated with backfat thickness in Duroc pigs by large-scale genome-wide association analysis. J Anim Sci. 2022;100(2):skac012. 10.1093/jas/skac012. 35034121 10.1093/jas/skac012 PMC8867564

[58] Calta J, Zadinová K, Čítek J, Kluzáková E, Okrouhlá M, Stupka R, et al. Possible effects of the MC4R Asp298Asn polymorphism on pig production traits under ad libitum versus restricted feeding. J Anim Breed Genet. 2023;140(2):207–15. 10.1111/jbg.12751. 36583444 10.1111/jbg.12751

[59] Bruun CS, Jørgensen CB, Nielsen VH, Andersson L, Fredholm M. Evaluation of the porcine melanocortin 4 receptor (MC4R) gene as a positional candidate for a fatness QTL in a cross between Landrace and Hampshire. Anim Genet. 2006;37(4):359–62. 10.1111/j.1365-2052.2006.01488.x. 16879346 10.1111/j.1365-2052.2006.01488.x

[60] Vergoni AV, Bertolini A. Role of melanocortins in the central control of feeding. Eur J Pharmacol. 2000;405(1–3):25–32. 10.1016/S0014-2999(00)00538-0. 11033311 10.1016/s0014-2999(00)00538-0

[61] Schneider JE. Energy balance and reproduction. Physiol Behav. 2004;81(2):289–317. 10.1016/j.physbeh.2004.02.007. 15159173 10.1016/j.physbeh.2004.02.007

[62] Yang F, Zhao S, Wang P, Xiang W. Hypothalamic neuroendocrine integration of reproduction and metabolism in mammals. J Endocrinol. 2023. 10.1530/JOE-23-0079. 10.1530/JOE-23-0079 37561042

[63] Marie PJ, Haÿ E, Modrowski D, Revollo L, Mbalaviele G, Civitelli R. Cadherin-mediated cell–cell adhesion and signaling in the skeleton. Calcif Tissue Int. 2014;94(1):46–54. 10.1007/s00223-013-9733-7. 23657489 10.1007/s00223-013-9733-7 PMC4272239

