# Supplementary figures and images for: Mendelian randomization and colocalization reveal potential causal effects of average daily gain on carcass composition and reproductive traits in pigs
Source: J Anim Sci Biotechnol. 2026 Apr 2;17:55. doi: 10.1186/s40104-026-01363-5 (PMC13045043; doi:10.1186/s40104-026-01363-5)

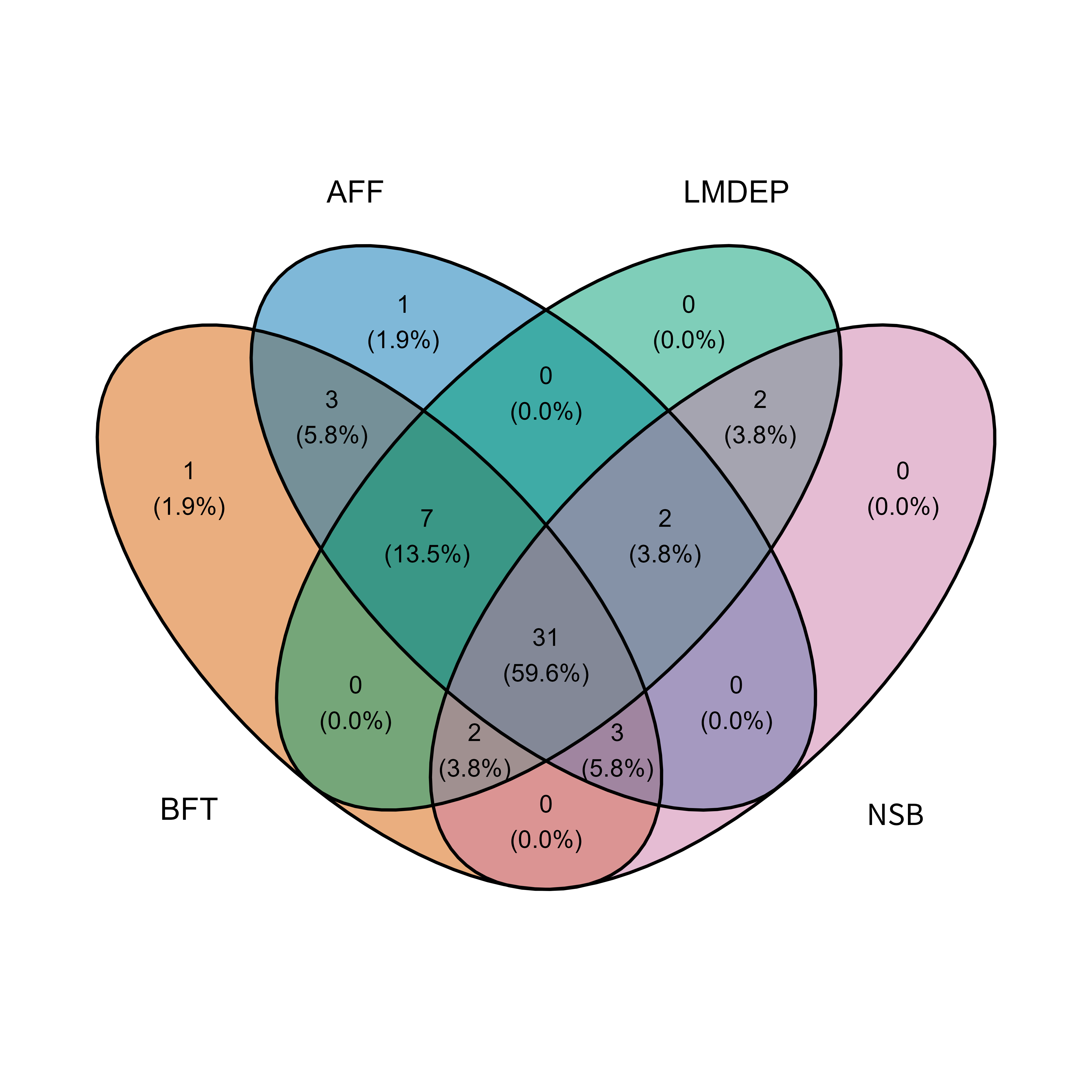

Supplement: Supplementary file 2 — Additional file 2: Fig. S1. Venn diagram of shared candidate genes identified through MR analyses between ADG and carcass composition traitsand reproductive traits. [file 40104_2026_1363_MOESM2_ESM.tif]

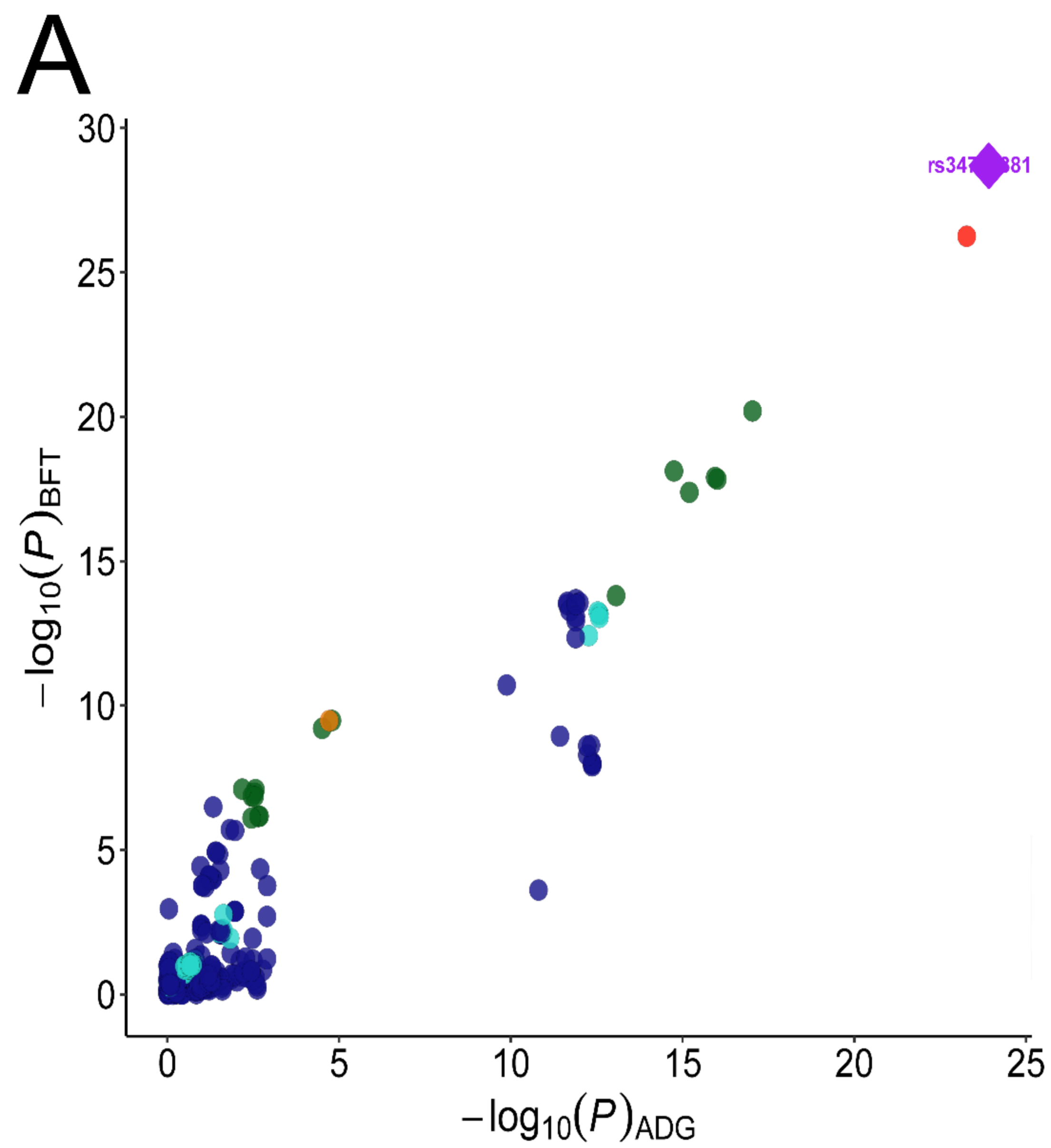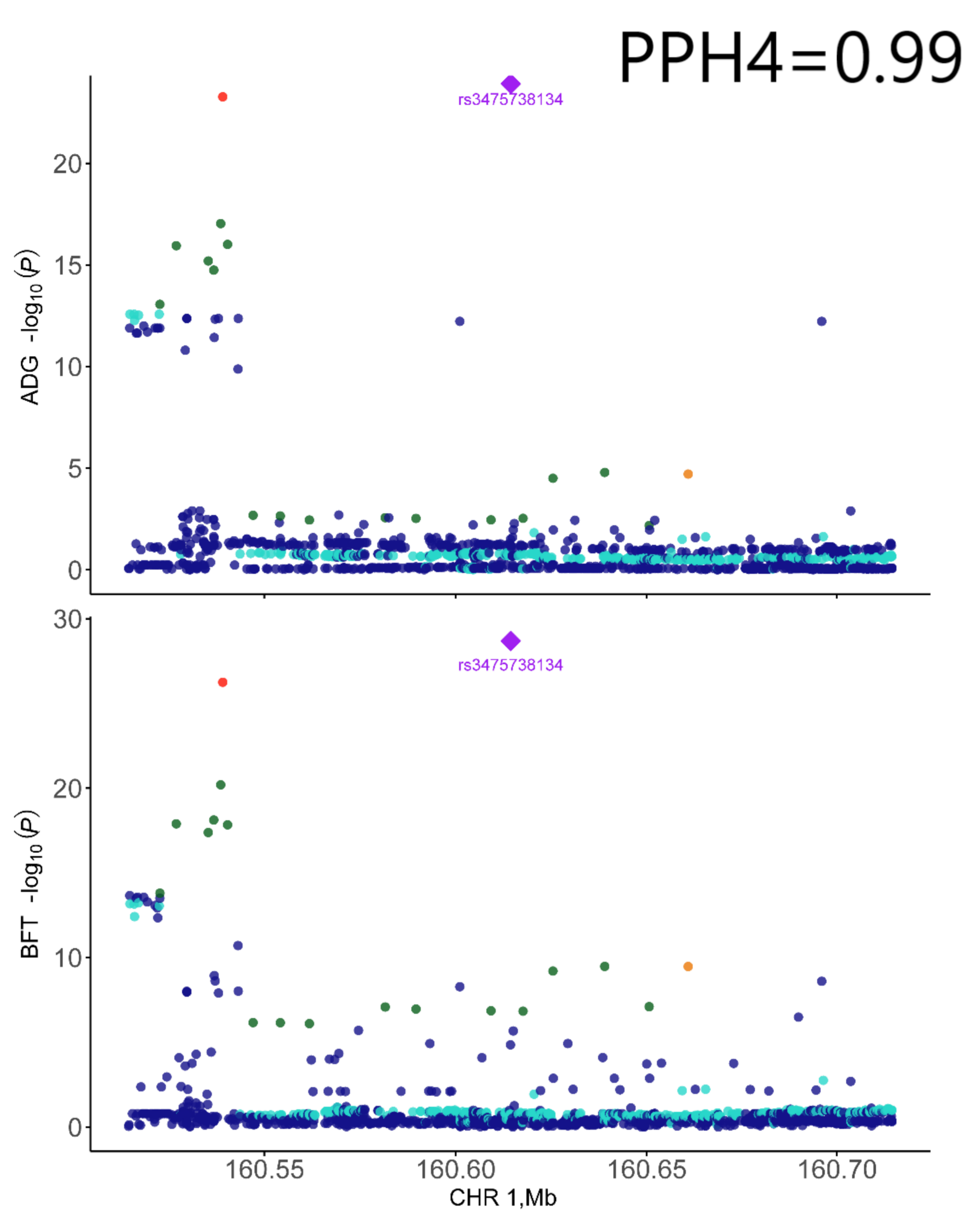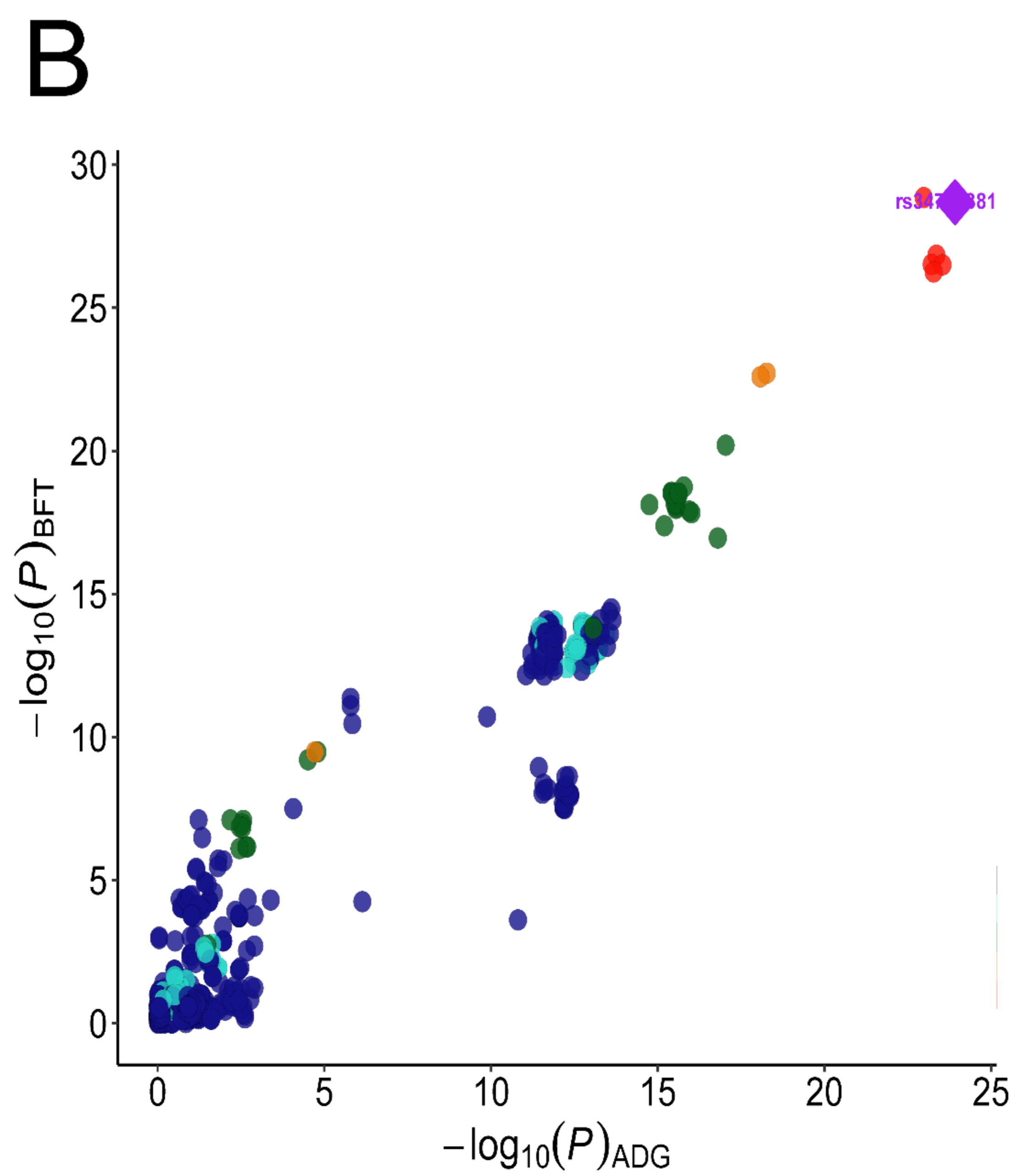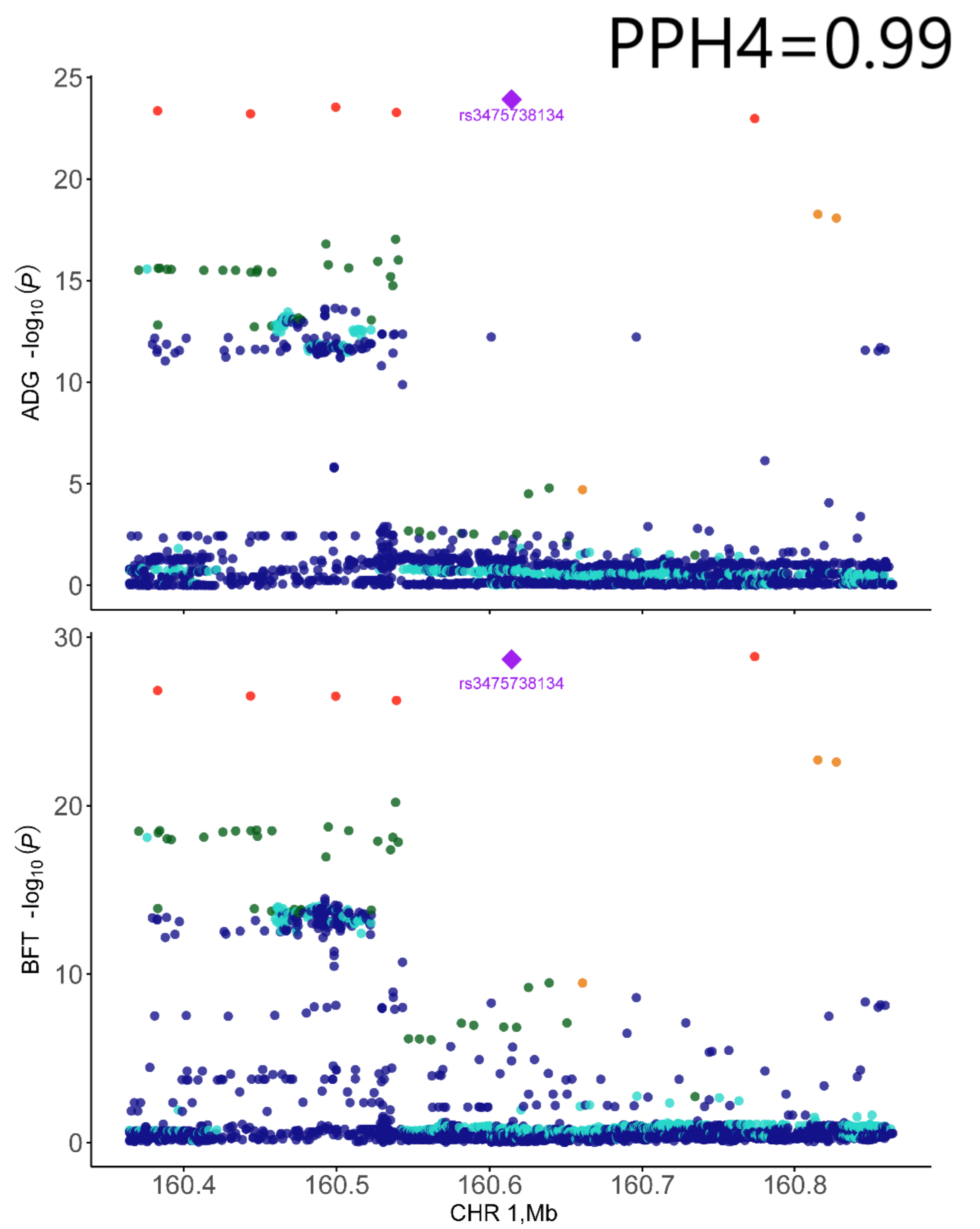

C

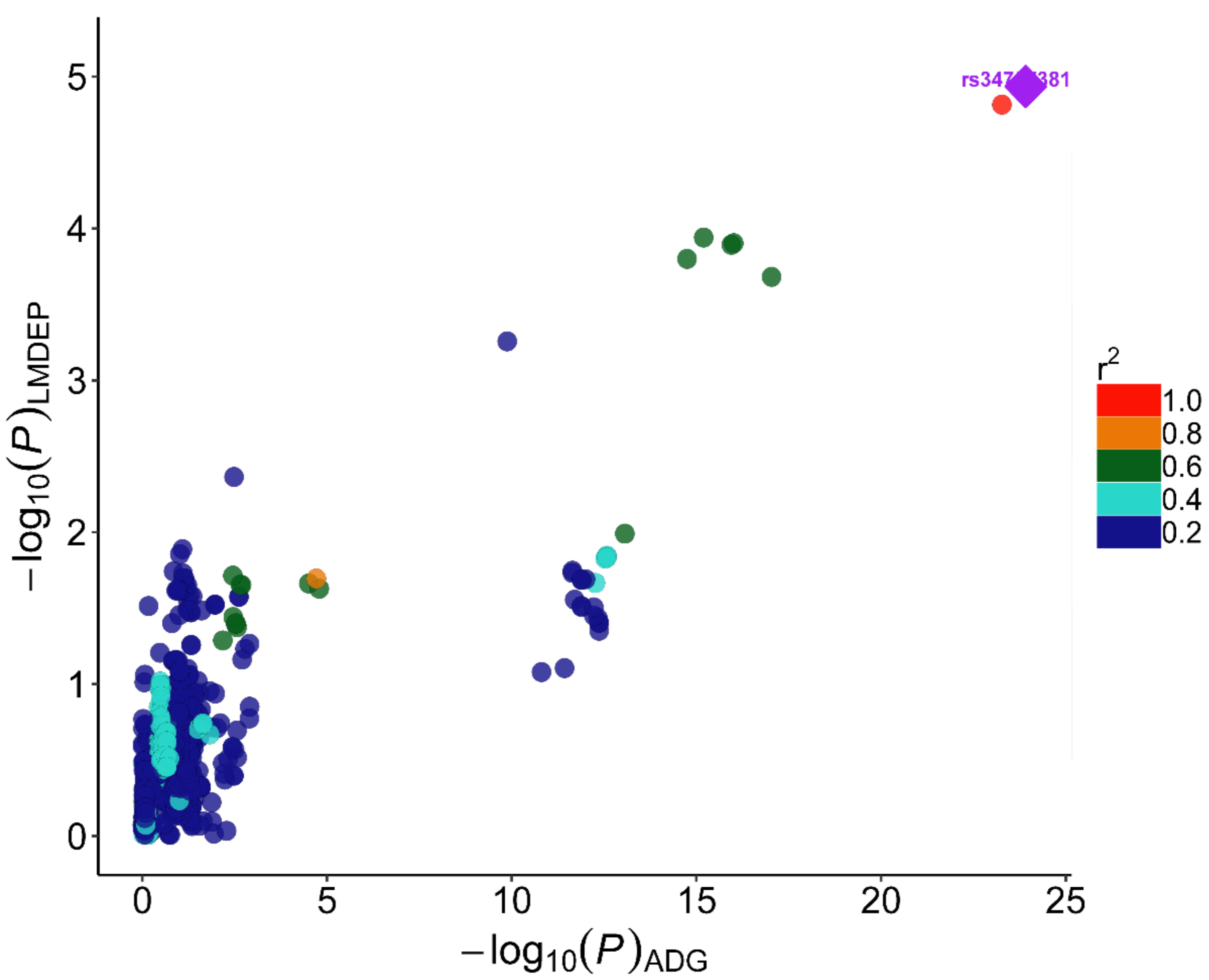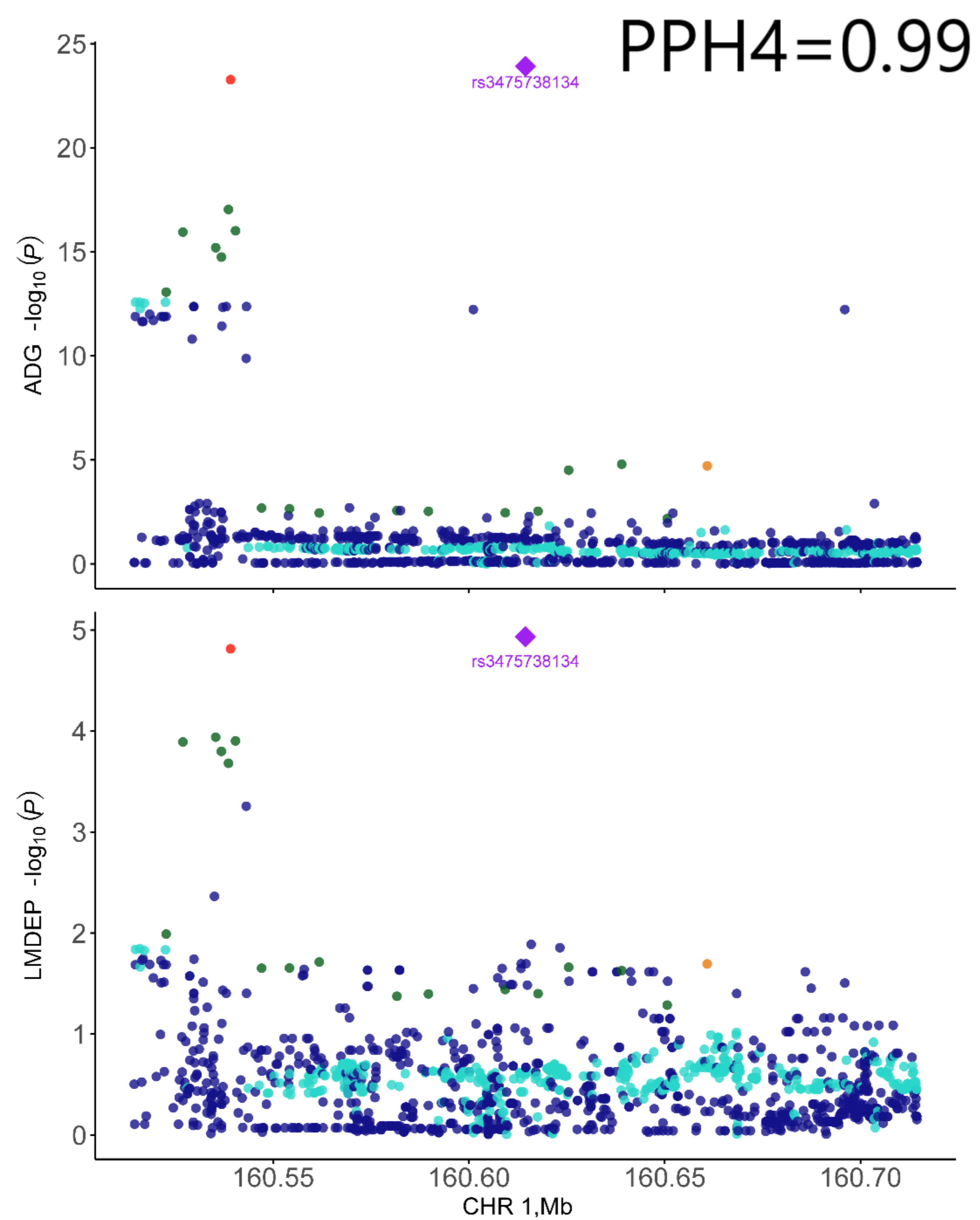

D

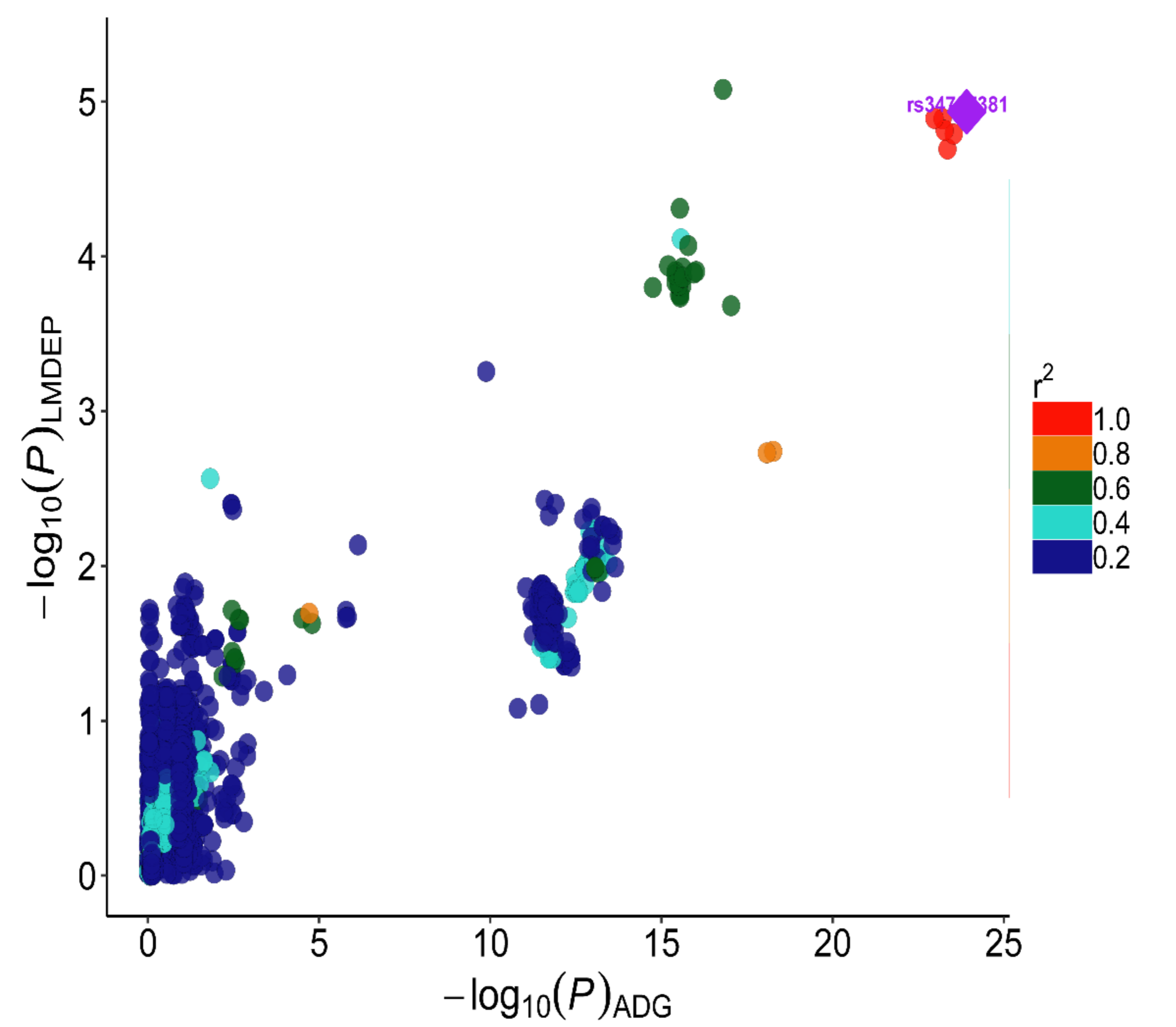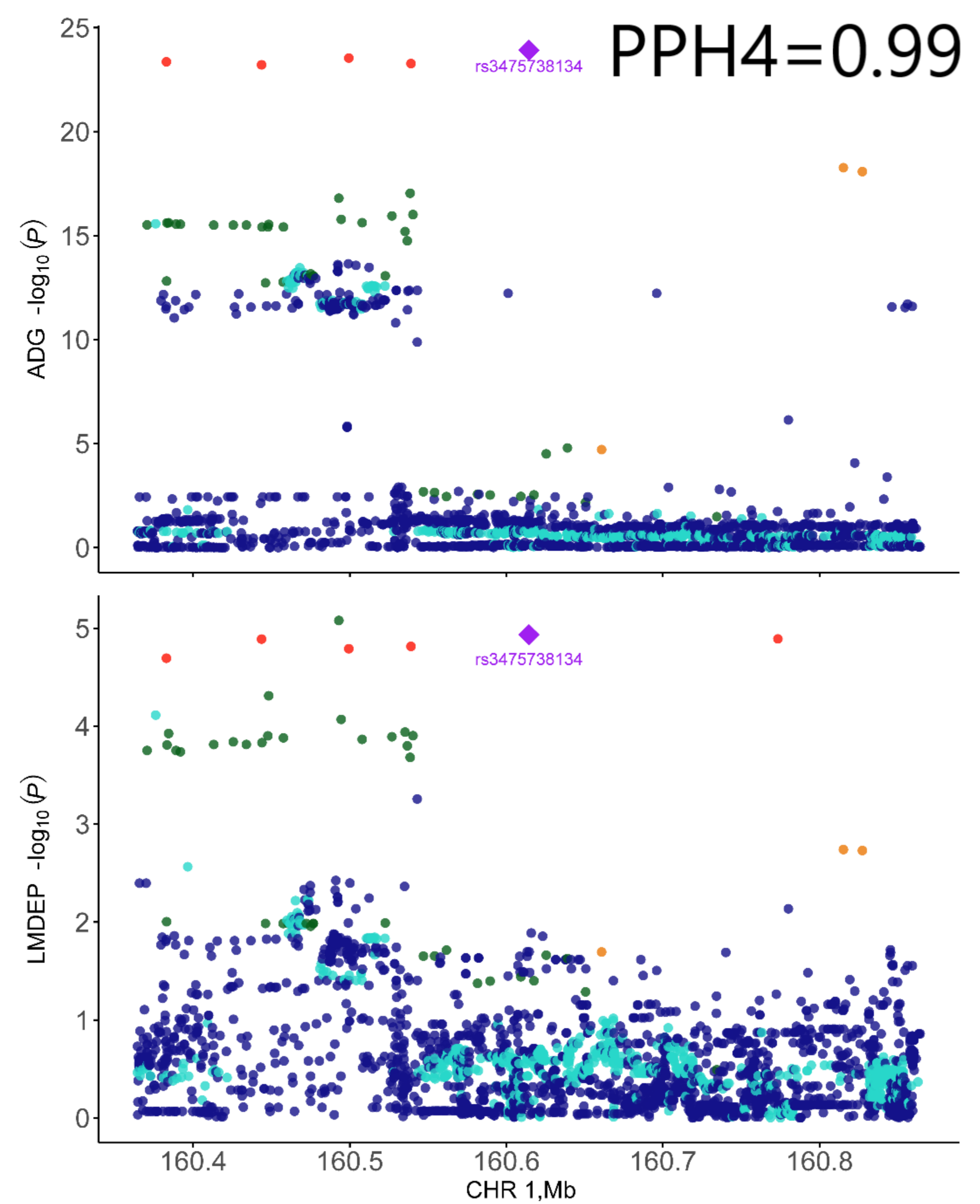

Supplement: Supplementary file 3 — Additional file 3: Fig. S2. Local Manhattan plots illustrate the colocalization between ADG and carcass composition traits using different genomic window sizes. A Colocalization between ADG and BFT within a ±100 kb window centered on the top colocalized SNP. B Colocalization between ADG and BFT within a ±250 kb window. C Colocalization between ADG and LMDEP within a ±100 kb window. D Colocalization between ADG and LMDEP within a ±250 kb window. For each panel, the left plot displays GWAS association signals for ADG and the corresponding carcass trait across the shared locus, while the right plot zooms into the specified window centered on the top colocalized SNP. SNPs are colored according to LD with the top colocalized SNP. [file 40104_2026_1363_MOESM3_ESM.pdf]

A

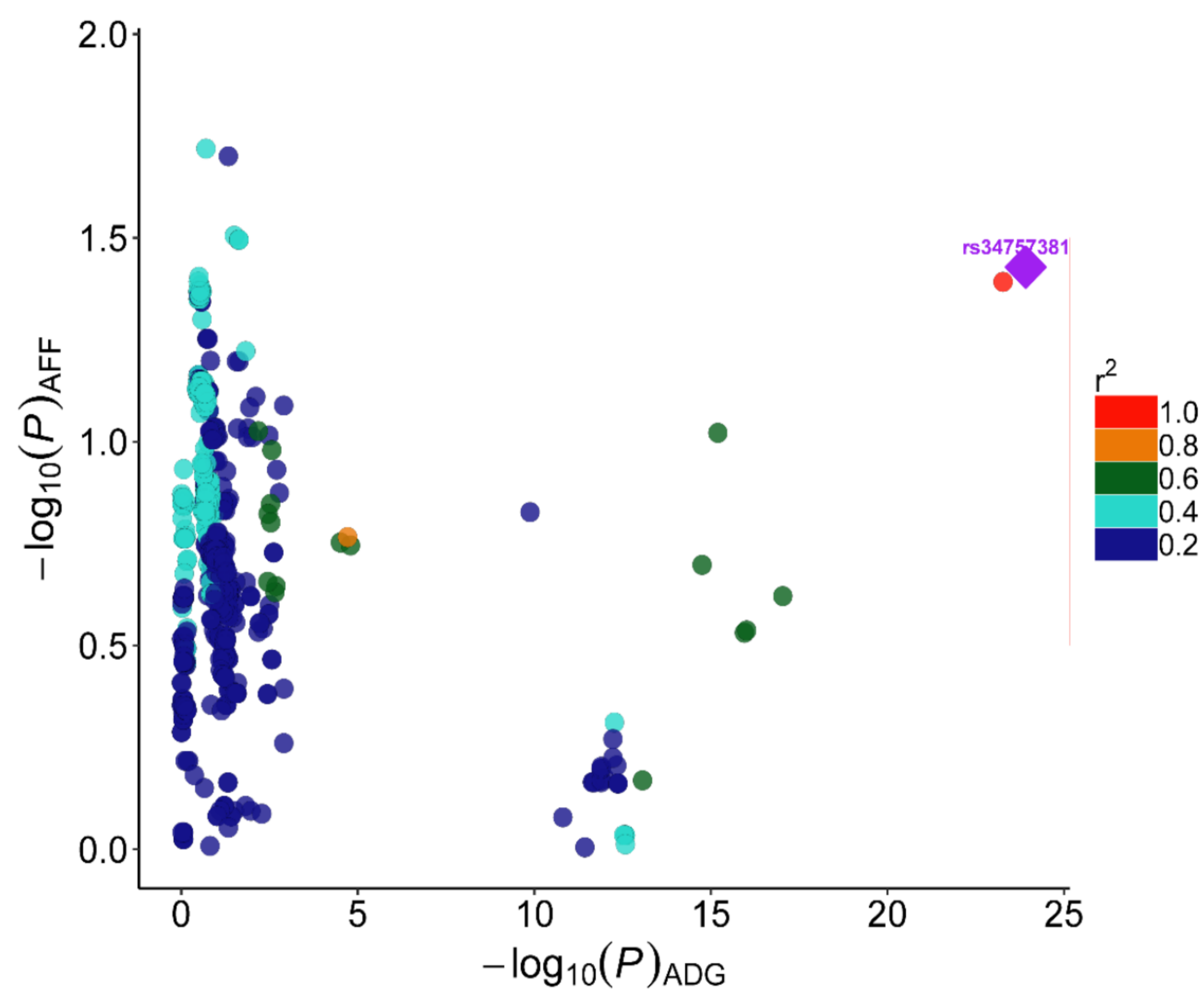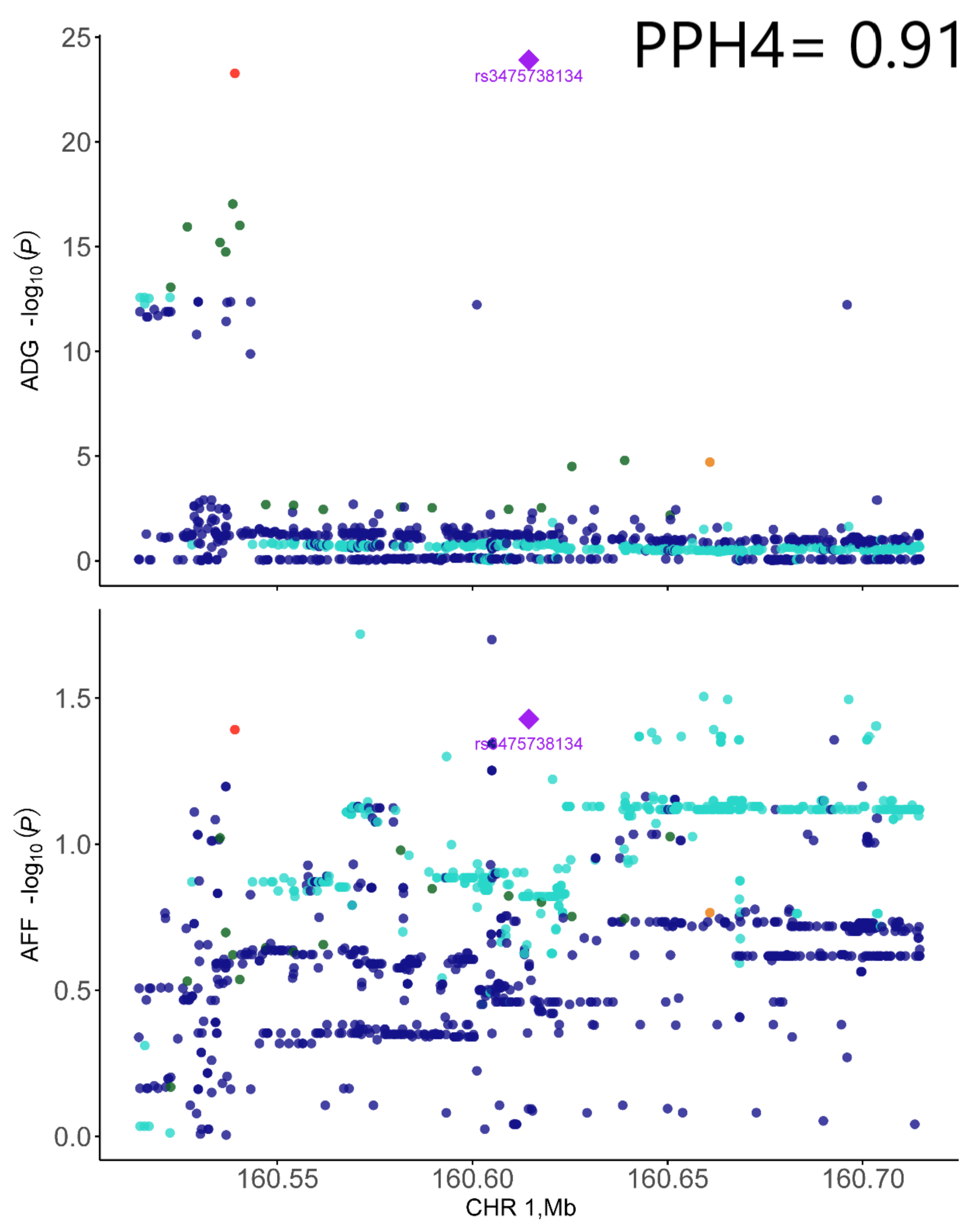

B

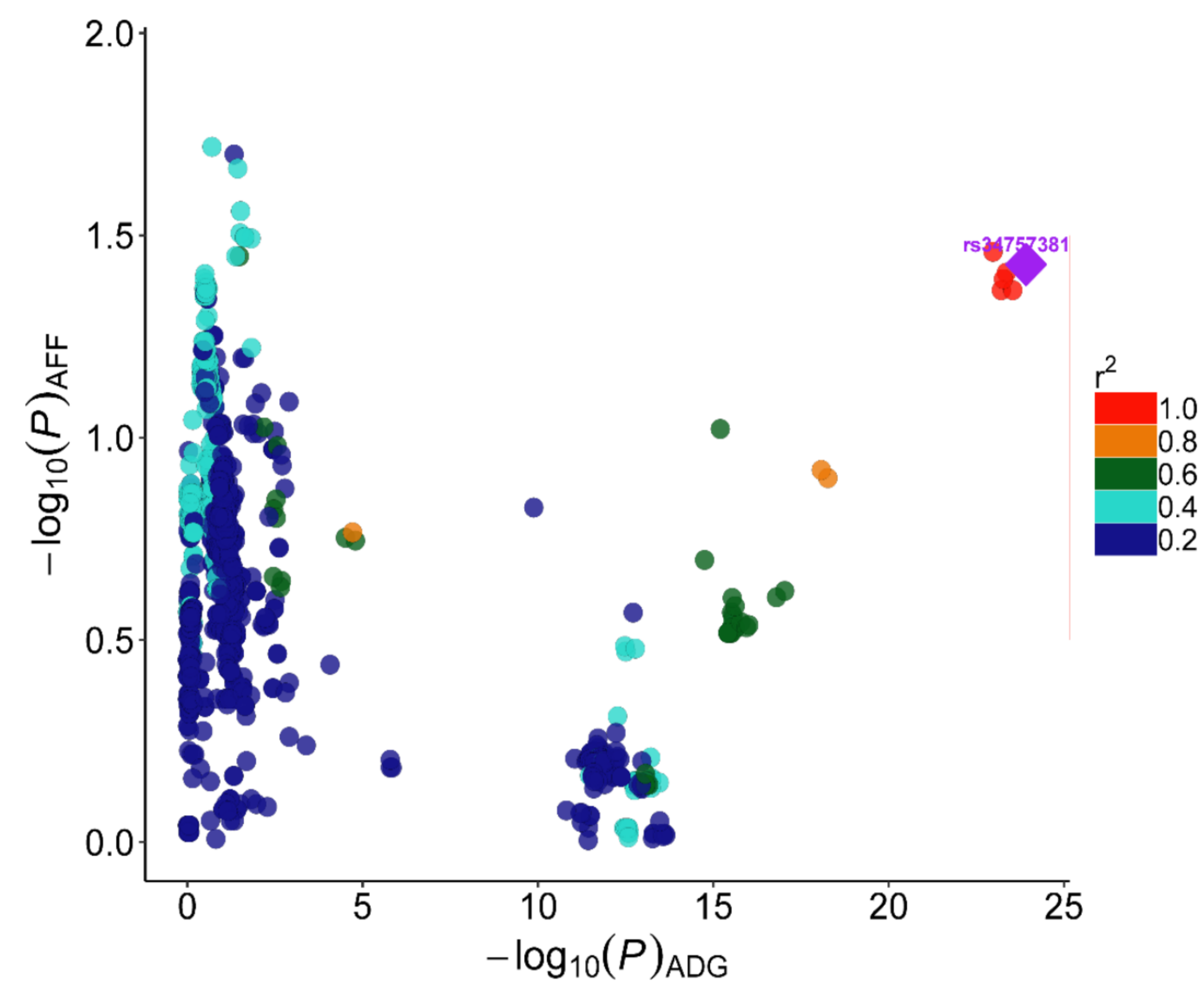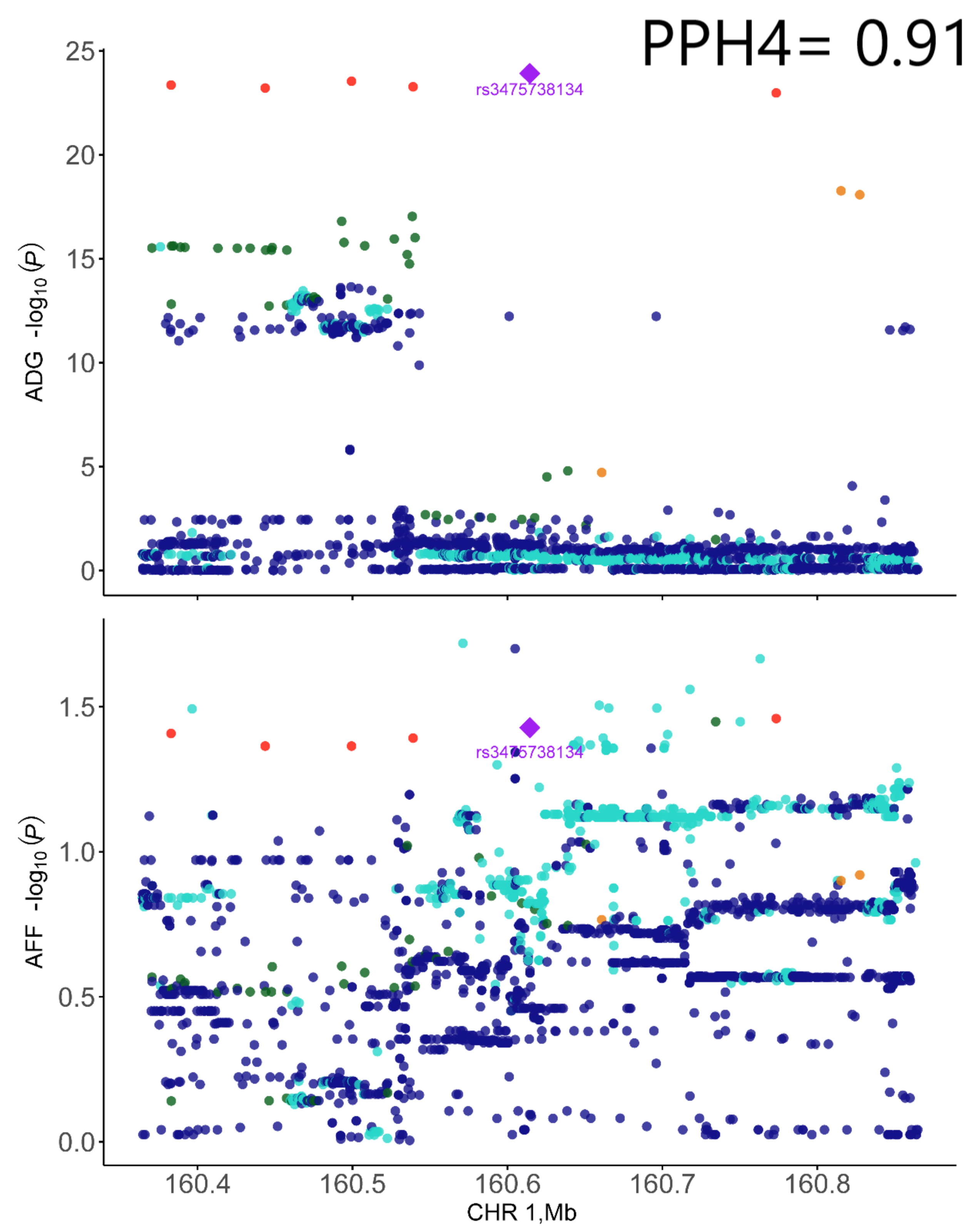

C

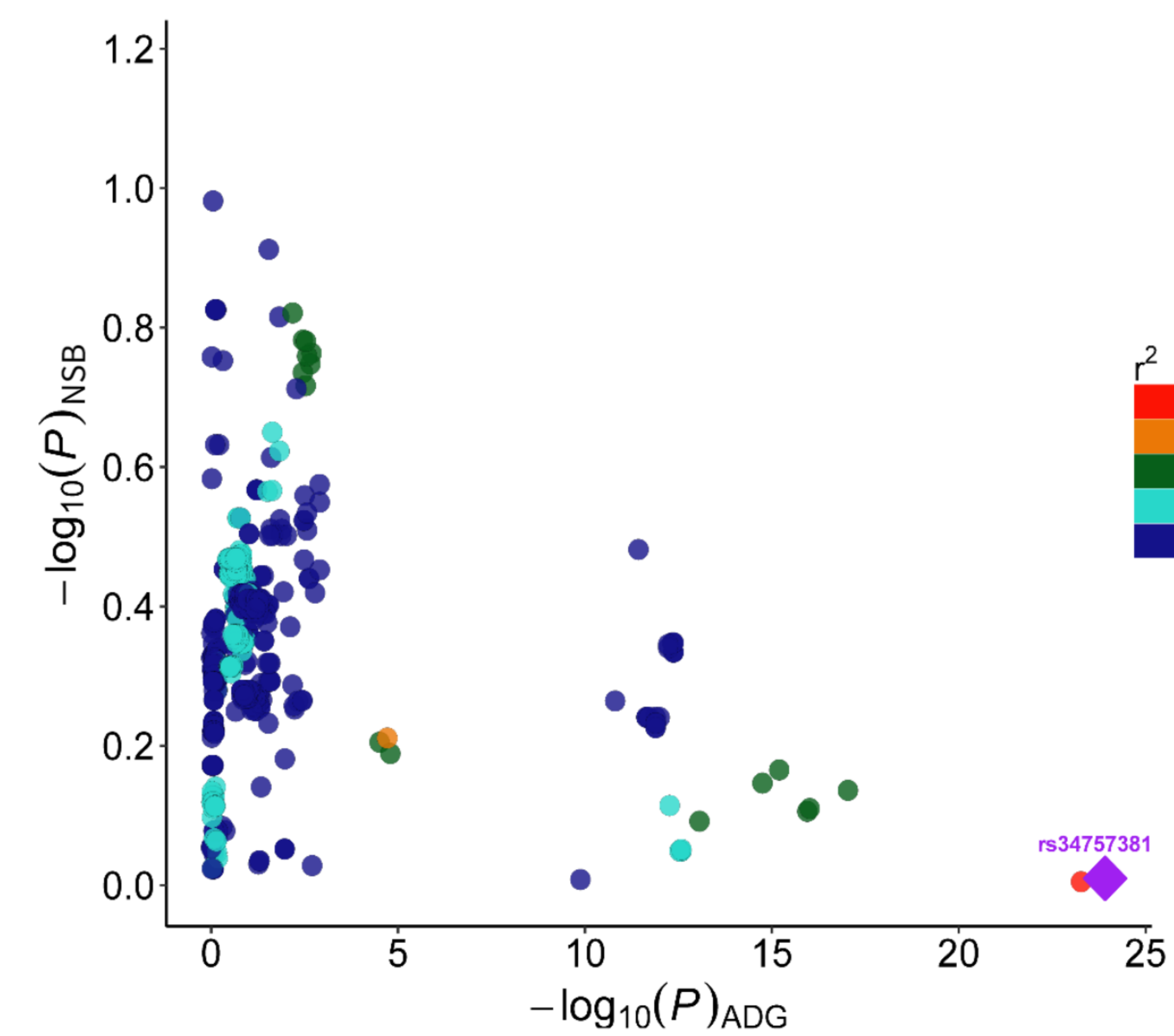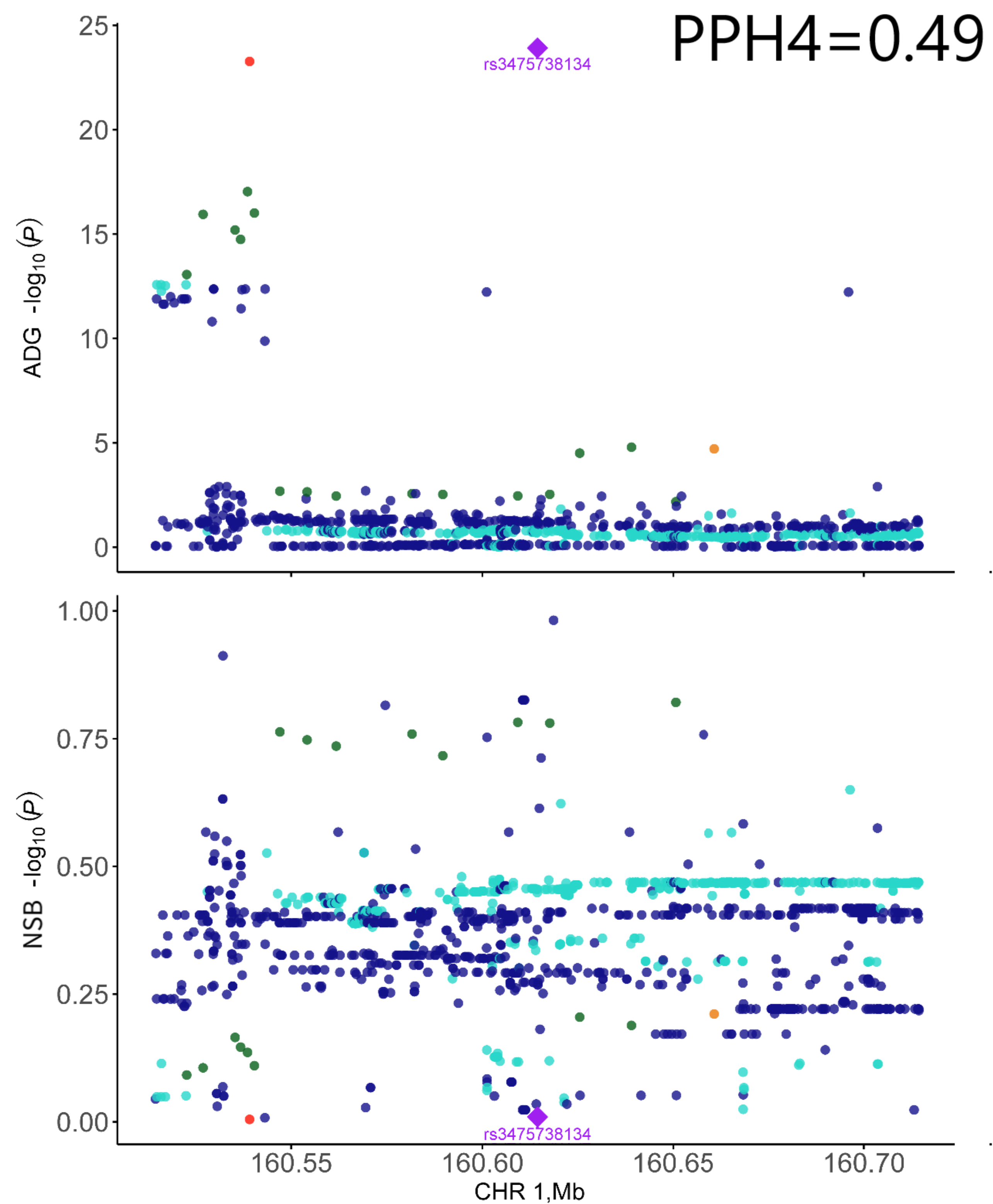

D

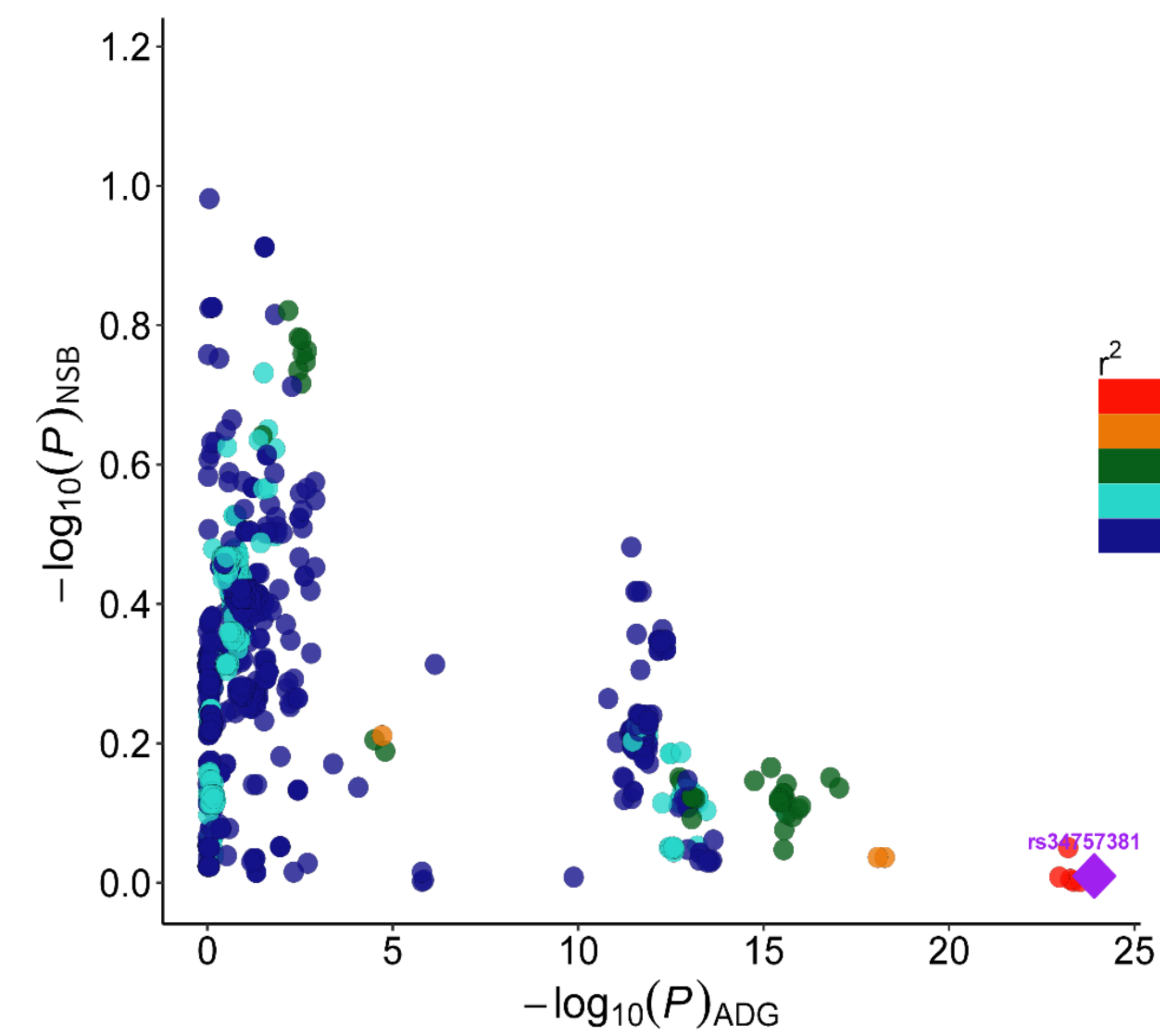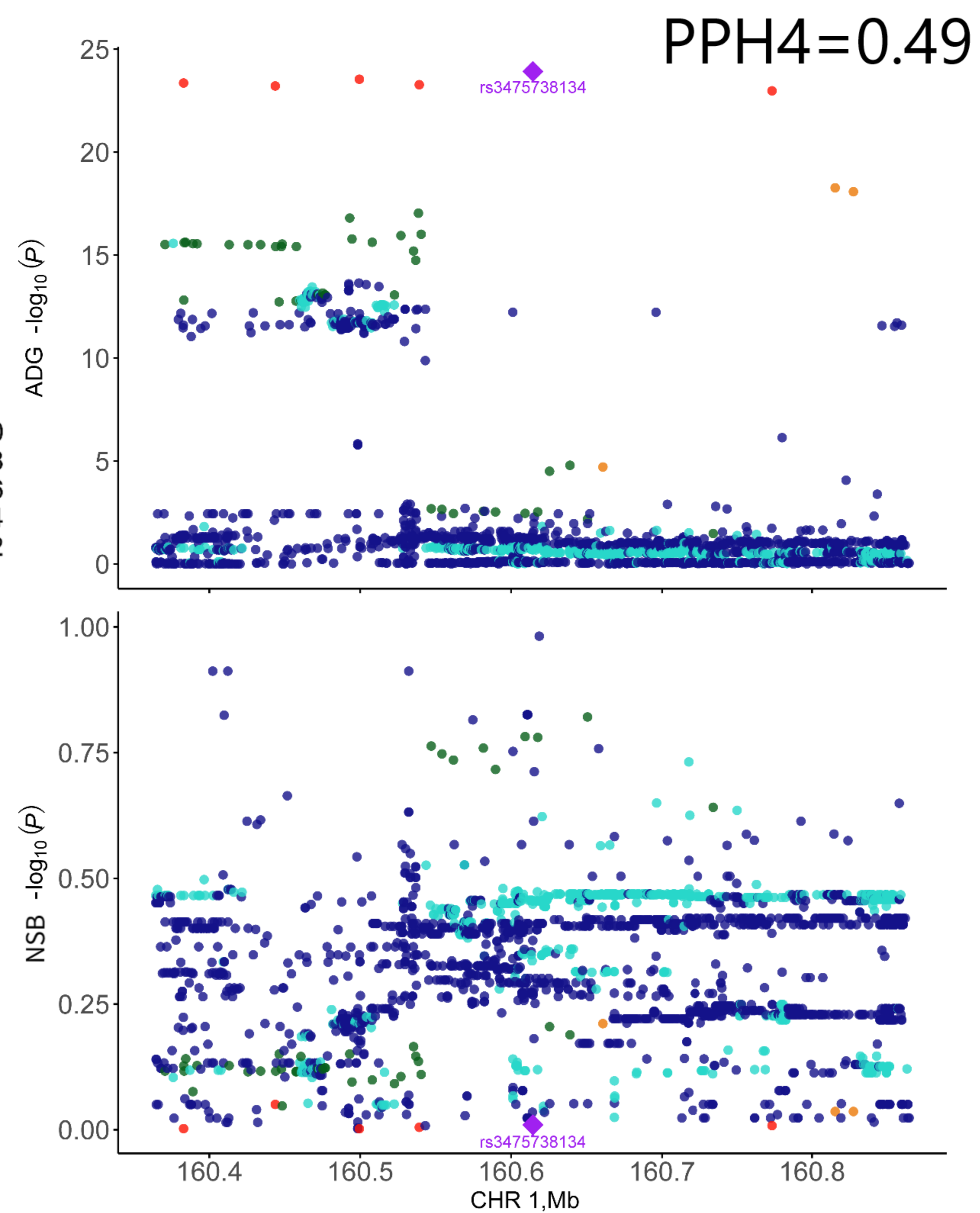

Supplement: Supplementary file 5 — Additional file 5: Fig. S4. Local Manhattan plots illustrate the colocalization between ADG and reproductive traits using different genomic window sizes. A Colocalization between ADG and AFF within a ±100 kb window around the lead colocalized SNP. B Colocalization between ADG and AFF within a ±250 kb window. C Colocalization between ADG and NSB within a ±100 kb window. D Colocalization between ADG and NSB within a ±250 kb window. For each panel, the left plot shows the GWAS association signals for ADG and the corresponding reproductive trait across the locus, while the right plot zooms into the specified window centered on the top colocalized SNP. The purple diamond denotes the top colocalized SNP, and the color scale indicates LD relative to this SNP. [file 40104_2026_1363_MOESM5_ESM.pdf]
